# Random auditory stimulation during sleep disturbs traveling slow waves and declarative memory

**DOI:** 10.1016/j.isci.2026.116601

**Published:** 2026-06-25

**Authors:** Nora M. Roüast, Deniz Kumral, Steffen Gais, Monika Schönauer

**Affiliations:** 1 Institute of Psychology, Neuropsychology, University of Freiburg, Freiburg im Breisgau, Germany; 2 BrainLinks-BrainTools, University of Freiburg, Freiburg im Breisgau, Germany; 3 Institute of Medical Psychology and Behavioral Neurobiology, University of Tübingen, Tübingen, Germany; 4 Bernstein Center Freiburg, University of Freiburg, Freiburg im Breisgau, Germany

**Keywords:** sleep, memory consolidation, auditory stimulation, traveling waves, SWS, declarative memory

## Abstract

Sleep plays a crucial role in memory consolidation, and various methods have attempted to enhance this process by using auditory stimulation. However, the broader impact of auditory stimulation on sleep physiology and memory retention is not fully understood. Here, we apply random or sham auditory stimulation during an afternoon nap to investigate its effects on neural activity and memory consolidation. Stimulation led to a specific reduction in slow-wave sleep, a decreased slow-wave count, and impaired declarative recall that correlated with the diminished sleep depth. Furthermore, we observed altered slow-wave traveling dynamics, with stimulation resulting in shorter traveling trajectories with less reach and reduced spatial spread, particularly impacting frontal regions. Disruptions in these features further predicted memory deficits. Our findings highlight the role of dynamic slow-wave properties in declarative memory consolidation and reveal some methodological limitations for using auditory cues during sleep, since they may disrupt complex processing patterns.

## Highlights

• Random auditory stimulation in nap disrupts slow-wave sleep and declarative memory
• Stimulation altered the traveling dynamics of slow waves detrimentally for memory
• Consolidation depended not only on occurrence but also the propagation of slow waves
• Stimulation can inadvertently interfere with hidden sleep-physiological processes

## Graphical abstract

**Figure:**
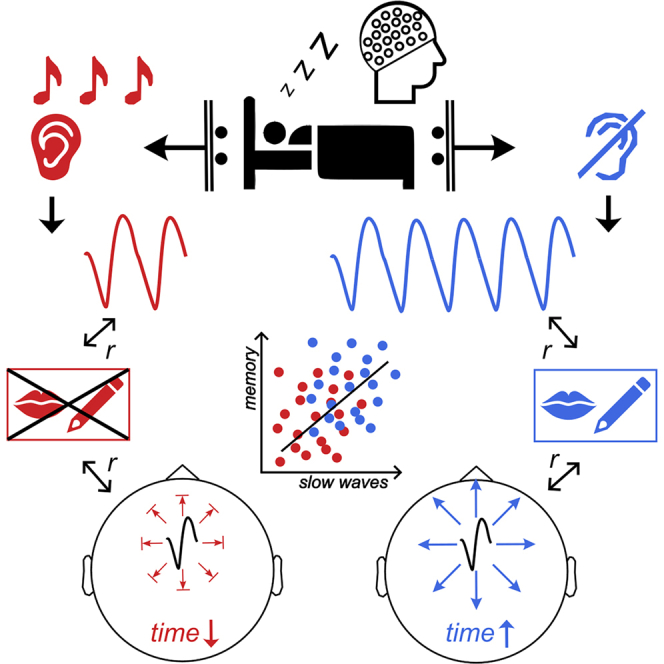

## Teaser

Health sciences; medicine; neuroscience

## Introduction

The dependence of memory consolidation on sleep has long been established for procedural learning,^1^ but seems particularly strong for declarative memory processing.^2^^,^^3^^,^^4^ According to contemporary models and backed by a growing body of research,^5^^,^^6^^,^^7^^,^^8^ covert reactivation of mnemonic content underlies sleep-dependent memory consolidation by propagating information from hippocampal to neocortical regions via a synchronized interplay of hippocampal sharp-wave ripples, thalamic spindles, and neocortical slow waves.^9^^,^^10^ In line with this idea, both spindle activity and slow waves have been related to memory consolidation in sleep.^11^^,^^12^^,^^13^ Indeed, reactivation drives memory retention^14^ and manipulation of the key oscillatory mechanisms that support this process modulates memory performance.^15^ Therefore, the relationship between memory reactivation and sleep physiology presents a potential avenue for improving memory consolidation.

Targeted memory reactivation has successfully utilized auditory or olfactory cues during sleep to improve later memory retention in humans.^16^ By presenting mnemonic cues linked to previous learning material, the re-emergence of specific learning-related activity patterns can be triggered during sleep such that this information gets preferentially consolidated. Moreover, the general opportunity for memory consolidation could be increased by boosting the supportive physiology, such as slow waves. Both transcranial magnetic and auditory stimulation were used to trigger or enhance slow waves.^13^^,^^17^^,^^18^ The timing of this auditory stimulation appears to be crucial, as cues synchronized with the slow-wave upstate enhance slow-wave activity and may consequently improve memory retention.^13^^,^^18^ Cues applied during identified slow waves irrespective of phase may lead to their suppression, even though the effects on memory are still unclear.^19^ Importantly, though, noise generally has been associated with detrimental effects on sleep depth, even if the overall sleep duration remains unchanged.^4^^,^^19^ Thus, auditory input during sleep can both lead to functional gains or losses, with the ultimate memory benefit of sleep potentially being the sum of both influences and depending on ties to content or timing.

Importantly, the mere occurrence of slow waves does not guarantee intact physiological properties, such as their traveling trajectory. Traveling slow waves allow specific states of brain connectivity to emerge, which serve memory processing.^20^^,^^21^^,^^22^^,^^23^^,^^24^ Yet, the traveling behavior of slow waves may depend on undisturbed sleep, with sounds being observed to alter the dynamic propagation of slow waves.^25^ Disturbing traveling behavior and therefore natural information propagation across the brain might consequently deteriorate memory consolidation.

Here, we aimed to investigate how randomly occurring sounds, unrelated to prior learning material and not synchronized with the slow wave phase, influence sleep-dependent memory consolidation and alter sleep-physiological processes crucial for memory processing. We expected that randomly administered auditory stimulation would disrupt deep sleep, the emerging dynamic properties of sleep physiology, and consequently mnemonic processing. Twenty healthy young adults underwent random auditory stimulation during sleep in a within-subject electroencephalography (EEG) study. They studied both procedural and declarative learning tasks before a nap opportunity that once included random auditory stimulation and once sham stimulation (silence). Auditory stimulation during sleep impaired declarative memory retention, led to a marked decrease in slow wave sleep (SWS) as well as slow wave activity, and stunted the traveling profiles of occurring slow waves. These affected markers of sleep physiology were each significantly related to the observed memory deficits.

## Results

**Figure 1:**
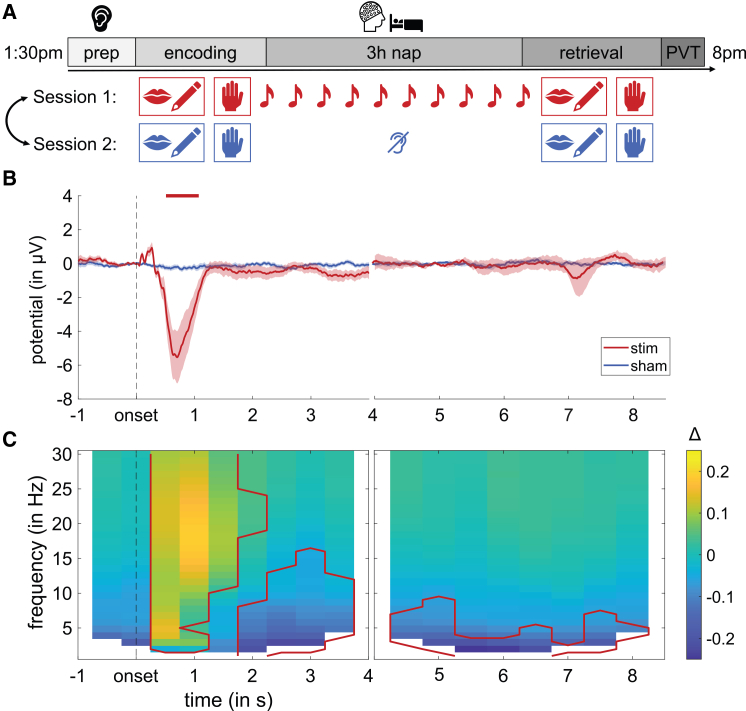
Experimental procedure and auditory stimulation (A) After a preparation phase, including hearing tests and EEG setup, participants encoded the declarative (learning-and-memory test, LGT-3) and procedural (sequential finger tapping) memory content. During a 3-h nap opportunity, EEG was recorded, and sounds were presented every 9.53 s in the stimulation session (red), but not in the sham session (blue). Subsequently, declarative and procedural memory were retrieved. Lastly, a psychomotor-vigilance test (PVT) was performed. The order of stimulation and sham sessions was randomly counterbalanced across participants. (B) Stimulation triggered a negative event-related potential (ERP). Plot displays average response in frontal channels (Fp1, Fp2, F3, Fz, F4, FC5, FC1, FC2, FC6, C3, Cz, C4, CP5) post sound onset. Time of significant difference according to permutation-based statistics is shown as a red bar. No ERP differences occurred in the consecutive segments (temporally continuous but separated only as a byproduct of preprocessing). Parallel processing resulted in a 100-ms baseline at around 5 s. Lines and shaded area denote mean ± SEM. (C) Time-frequency (TF) analyses contrast stimulation and sham condition across all channels show a broad band stimulation-triggered increase (yellow hues), followed by a decrease in lower frequencies (dark blue). Red outline denotes significance (*p* < 0.05). The decreased power in low frequencies in the stimulated versus the sham condition also remained in the consecutive but temporally continuous segments without stimulation. Plots B and C present continuous time but do not necessarily comprise the same trials, with segmentation in artifact rejection leading to separate time windows. All statistical analyses displayed were non-parametric cluster-based permutation tests performed on a dataset of 40 naps total (*n* = 20 with two sessions each).

Twenty healthy participants visited the sleep lab for two experimental sessions, during which they encoded declarative and procedural memory content before a 3 h nap opportunity, and retrieved the information subsequently (Figure 1A). In the stimulation session, sounds were played throughout the nap opportunity, whilst in the sham session, no sounds were presented. EEG recordings during the nap period, as well as retrieval performance, served as markers of the effect of stimulation on sleep quality and physiology, as well as the potential behavioral cost.

### Stimulation reduced slow-wave sleep, but left sleep duration and general alertness mostly intact

**Figure 2:**
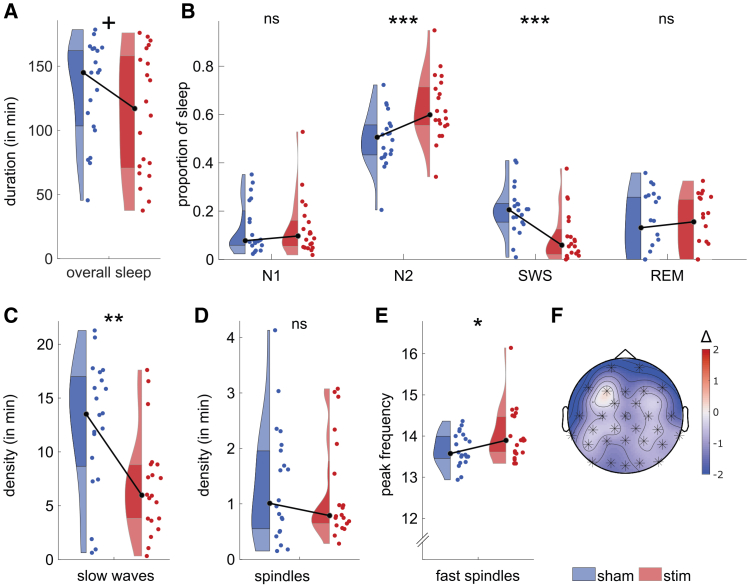
Impact of auditory stimulation on sleep physiology (A) Sleep duration (in seconds) in sham (shown in blue) and stim conditions (shown in red). Auditory stimulation marginally but not significantly decreased total sleep duration. (B) Proportion of time spent in each sleep stage, relative to the total duration. Stimulation significantly reduced SWS and increased N2 proportion. (C) Slow wave density by minute in established NREM sleep, averaged across all channels, was reduced for the stimulation condition. (D) Average spindle density by minute in established NREM sleep did not differ between conditions. (E) Stimulation was associated with increased peak frequency of fast spindles. (F) The topography contrast (stim-sham) showed a global decrease in delta power in response to stimulation. Violin plots show median values as a black dot and quartiles as a darker shaded area on the left side, as well as condition means of the individual participants as dots on the right-hand side. Statistical analyses displayed consisted of paired t-tests (A, B SWS + REM, C, D), Wilcoxon signed-rank tests (B N1 + N2, E), and non-parametric cluster-based permutation tests (F), performed on a dataset of 40 naps total (*n* = 20 with two sessions each). Significance levels are denoted as follows: ∗∗∗ <0.001, ∗∗ <0.01, and ∗ <0.05, + < 0.1.

We first compared sleep duration, relative time spent in sleep stages, and vigilance measures to evaluate whether auditory stimulation impacted sleep. We observed that the stimulation only marginally decreased sleep duration, measured in epochs scored as asleep (*t*_19_ = 1. 91, *p* = 0.071, *d* = 0.43; Figure 2A), and did not decrease the duration between first falling asleep and last waking (*t*_19_ = 1.28, *p* = 0.217, *d* = 0.29). However, it significantly reduced the time spent in SWS, both in absolute number of minutes spent in SWS and relative to the total sleep duration (*t*_19_ = 5.37, *p* < 0.001, *d* = 1.20 and *t*_19_ = 5.95, *p* < 0.001, *d* = 1.33; Figure 2B). Instead, participants in the stimulated nap condition spent relatively more time in light non-REM sleep stage N2 (*W*_19_ = 200, *p* < 0.001, *r* = 0.79), even though this effect was not significant for the absolute minutes of sleeping time (*t*_19_ = −0.78, *p* = 0.447, *d* = −0.17).

The duration of time spent in N1 and REM stages of sleep did not differ across stimulation conditions (*p* > 0.620, Table S1). The effects were not due to a delay in falling asleep or fragmented sleep, given that neither sleep latency nor time awake within the sleep period differed across stimulation conditions (see S1). None of these effects could be explained by the different sessions, given the absence of order effects (Table S2). Therefore, while auditory stimulation did not prevent subjects from falling asleep or maintaining sleep, it specifically reduced SWS, which was compensated by an increase in lighter N2 sleep. This altered the overall sleep composition while retaining a similar duration. Alertness levels were not affected by the reduction in sleep depth: There was no significant difference in the PVT between the stimulation (*M* = 298.61, *SD* = 24.85, *SE* = 5.56) and sham conditions (*M* = 302.21, *SD* = 25.69, *SE* = 5.74; *t*_19_ = −0.98, *p* = 0.338, *d* = 0.22), nor between the first (*M* = 297.56, *SD* = 25.02, *SE* = 5.59) and second experimental sessions (*M* = 303.27, *SD* = 25.32, *SE* = 5.66; *t*(19) = −1.62, *p* = 0.121, *d* = −0.36).

### Stimulation triggered a broad cortical response with a lasting low-frequency reduction

Since the overall pattern in sleep changed due to stimulation, we also assessed the immediate effects of the auditory stimulus on event-related potentials (ERPs) and trigger-bound alterations of the time-frequency trace (TF). Cluster-based permutation testing revealed a significant difference between ERP waves in the stimulation and the sham condition after sound onset, with a frontal cluster (Fp1, Fp2, F3, Fz, F4, FC5, FC1, FC2, FC6, C3, Cz, C4, CP5) showing a negative inflection from 0.509 to 1.072 s post stimulus (*p* = 0.027, Figure 1B) and a posterior cluster (TP9, TP10, P7, P8, PO9, O1, Oz, O2, PO10) showing a positive inflection between 0.375 and 0.951 s post stimulus (*p* = 0.032). The temporally consecutive segment showed no ERP effect between stimulation and sham conditions (*p* > 0.640, Figure 1B). It should be noted that features of the triggered individual ERP waves varied across participants and appeared to be more pronounced during NREM than REM stages, perhaps unsurprisingly, since sound stimuli presented in sleep are known to induce k complexes (Figures S1 and S2). Further, validation analyses using mastoid referencing instead of a common average reference produced equivalent results with a strong triggered ERP post stimulation only (Figure S3). The stimulation-triggered ERP was matched by the TF analyses, where the stimulation condition showed a significant increase in a broad frequency range (2–30 Hz) and all channels from 0.5 to 2 s post stimulus (*p* < 0.001, Figure 1C). Consecutively, 2–3.5 s post stimulus, there was a significant decrease (*p* = 0.019) in lower frequencies (1–16 Hz) across almost all channels (Fp1, Fp2, F7, F3, Fz, F4, F8, FC1, FC2, T7, C3, Cz, C4, T8, TP9, CP5, CP1, CP2, CP6, TP10, P7, P3, Pz, P4, P8, PO9, O1, Oz, O2, PO10). The reduction in lower frequencies (1–9 Hz) observed in the stimulation condition persisted into consecutive time windows (*p* = 0.035, Figure 1C).

### Stimulation decreased physiological markers of slow waves but not sleep spindles

Having demonstrated a specific decrease in SWS in the face of stimulation-triggered disruption, we further investigated whether auditory stimulation altered the oscillatory markers involved in memory consolidation, namely slow waves and sleep spindles. Fewer individual slow waves were detected across all channels for the entire nap duration for the stimulation condition (*M* = 17194.24, *SE* = 2193.24), relative to the sham condition (*M* = 34267.90, *SE* = 3912.03; *t*_19_ = 3.83, *p* = 0.001, *d* = 0.86). This effect persisted when considering slow wave count during epochs identified as established NREM (N2–N4, henceforth “NREM sleep”) only (*t*_19_ = 3.92, *p* < 0.001, *d* = 0.88). Slow wave density by minute and channel within NREM sleep was significantly reduced in the stimulated condition (*M* = 6.94, *SE* = 1.07) relative to the sham condition (*M* = 12.42, *SE* = 1.38; *t*_19_ = 3.55, *p* = 0.002, *d* = 0.79; Figure 2C). Even within SWS, stimulation was associated with significant detriment to slow wave density (*t*_19_ = 2.81, *p* = 0.011, *d* = 0.63). Interestingly, there was no difference in mean amplitude of identified slow waves across conditions (*W*_19_ = 76, *p* = 0.294, *r* = 0.24), indicating that any resulting condition differences in SWS were not due to slow waves remaining below the amplitude criterion. In contrast, spindles showed no such detriment of stimulation with respect to count or density within NREM sleep (count: *t*_19_ = 1.14, *p* = 0.270, *d* = 0.25; density: *t*_19_ = 0.60, *p* = 0.554, *d* = 0.13, Figure 2D). Even spindle density restricted to N2 did not differ across stimulation conditions (*t*_19_ = 1.42, *p* = 0.171, *d* = 0.32). However, a further exploratory investigation of potentially affected spindle properties did show a significant increase in peak spindle frequency for fast spindles in the stimulation condition (*M* = 14.02 Hz, median = 13.89, *SE* = 0.15) in contrast to the sham condition (*M* = 13.68 Hz, *median* = 13.57, *SE* = 0.09; *W*_19_ = 181.5, *p* = 0.004, *r* = 0.64; Figure 2E). No such frequency increase was observed for slow spindles (*W*_19_ = 78, *p* = 0.117, *r* = −0.35). There was also no difference across conditions for spindle duration (*p* = 0.162) or activity (*p* = 0.684). Likewise, phase-coupling analyses of identified slow waves and spindles showed no differences in mean phases or number of coupling events across conditions for frontal or posterior channels (Figures S4A, S4B, S4E, and S4F). However, auditory stimulation increased the proportion of slow wave-spindle coupling for both frontal and parietal regions, when taking the stimulation-dependent decrease of slow waves into account (Figures S4C and S4D), indicating that auditory stimulation increased the efficiency of slow wave-spindle coordination without increasing total coupling events or altering their temporal structure. A likely explanation for this finding is that detected slow waves in the stimulation condition contain a substantial fraction of stimulation-induced K-complexes with a coupled spindle. However, the efficiency of coupling did not relate to behavioral performance (*p* > 0.230).

The stimulation cost to slow waves but not spindles was confirmed in frequency analyses: Cluster-based permutation testing on broadband frequency data (0.5–30 Hz) revealed a significant negative cluster between stimulation and sham conditions (cluster-level statistic = −1730.1, *p* = 0.029), amounting to a significant reduction of power almost matching the delta frequency band (0.6–5.7 Hz). Analyses for frequencies of interest confirmed significant decreases in delta band (0.5–4 Hz) for the stimulation condition (cluster-level statistic = −41.70, *p* = 0.008, Figure 2F), while no condition difference was observed for spindle frequencies (12–16 Hz). Together, stimulation thus consistently affected sleep slow wave activity, reducing slow wave count and density, while leaving markers of spindle activity comparably intact.

### Stimulation disturbed the consolidation of declarative but not procedural memory content

**Figure 3:**
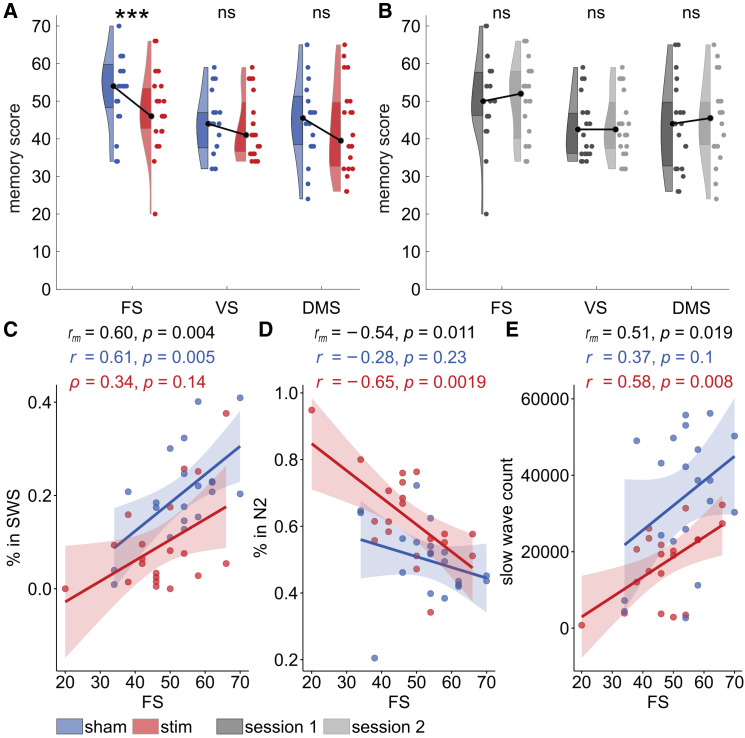
Effects of auditory stimulation on memory performance (A) Declarative memory performance, shown as figural (FS), verbal (VS), and overall declarative memory score (DMS) after the stimulation (red) and non-stimulation nap (blue). Stimulation significantly reduced the figural declarative memory score. (B) No difference emerged for declarative performance between the first and second experimental sessions, showing that there is no effect of repeated testing on the two different forms of the test. (C) Increased proportion of time in SWS was related to improved figural memory performance overall, and specifically in the non-stimulation condition. (D) Increased proportion of time in N2 was related to decreased figural memory performance overall, and specifically in the stimulation condition. (E) Higher count of identified slow waves was related to improved figural memory performance overall, and specifically in the stimulation condition. Dots indicate individual data points, and the line regression of each condition. Black statistics denote repeated measures correlations. Violin plots show median values as a black dot and quartiles as a darker shaded area on the left side, as well as condition means of the individual participants as dots on the right-hand side. Statistical analyses displayed consisted of paired t-tests (A and B) and correlations as denoted (C, D, and E: repeated measure correlations as r_rm_; Pearson’s as r; Spearman as ρ). Paired t-tests and repeated measure correlations were performed on the total dataset of 40 naps (*n* = 20 with two sessions each), with the other correlations being performed on the individual condition (*n* = 20). Significance levels are denoted as follows: ∗∗∗ <0.001, ∗∗ <0.01, and ∗ <0.05, + < 0.1.

Having confirmed the detrimental effect of auditory stimulation on the depth of NREM sleep, and specifically slow-wave activity, we further assessed whether stimulation impacted declarative and procedural memory consolidation. There was a significant decrease in declarative memory retention, particularly for visuospatial memory, after a nap with auditory stimulation (figural memory score *FS*: *M* = 47.3, *SE* = 2.42), compared to the sham condition (*M* = 53.4, *SE* = 2.27; *t*_19_ = 4.35, *p* < 0.001, *d* = 0.97; Figure 3A). However, we found no effect of stimulation on the verbal retention score VS (*t*_19_ = 0.36, *p* = 0.721, *d* = 0.08), or the overall declarative memory score *DMS* (*t*_19_ = 1.34, *p* = 0.196, *d* = 0.30; Figure 3A). Notably, there were no significant order effects on declarative memory performance between the first and the second testing sessions (FS: *t*_19_ = 0.15, *p* = 0.881, *d* = 0.04; VS: *t*_19_ = −0.19, *p* = 0.848, *d* = −0.04; DMS: *t*_19_ = −0.56, *p* = 0.581, *d* = −0.13; Figure 3B). In contrast to the detrimental effect on declarative memory processing, stimulation did not affect procedural memory performance, assessed in accuracy and speed of reproduced finger tapping sequences (Figure S5). Taken together, stimulation appeared to have a detrimental impact on visual-spatial memory alone, whilst leaving other parts of declarative or procedural memory intact. All subsequent analyses using memory performance focus on the figural score as part of the declarative memory performance.

### Disruptions in sleep physiology were related to the detriment of declarative memory performance

Considering the influence of auditory stimulation on sleep physiology and declarative memory performance, we tested whether the behavioral and physiological effects were related. Indeed, the differences in sleep architecture in response to stimulation correlated with the figural score, with repeated measures correlations showing a benefit of relative time spent in SWS and a cost of increasing time spent in N2 (*r*_*rm*_ = 0.60, *p* = 0.004, and *r*_*rm*_ = −0.54, *p* = 0.011; Figures 3C and 3D). Condition-specific correlations revealed that pattern to be driven by a benefit of relative time spent in SWS for the sham, *r* = 0.61, *p* = 0.005 (*ρ* = 0.52, *p* = 0.018), rather than the stimulation condition (*ρ* = 0.34, *p* = 0.139). For relative time spent in N2, the detrimental relationship with figural memory was significant for the stimulation condition but did not reach significance in the sham condition (*r* = −0.65, *p* = 0.002 and *r* = −0.28, *p* = 0.226, respectively). Furthermore, greater numbers of detected slow waves positively correlated with higher figural memory scores across conditions (*r*_*rm*_ = 0.51, *p* = 0.019). Separate analyses showed that this effect was significant for the stimulation but not the sham condition (*r* = 0.58, *p* = 0.008 and *r* = 0.37, *p* = 0.104, respectively; Figure 3E). Overall, the shift in sleep patterns was thus robustly related to behavioral performance, although not all individual condition-specific correlations reached significance.

### Traveling waves as a potential mechanism of propagating information and the limiting effects of stimulation

**Figure 4:**
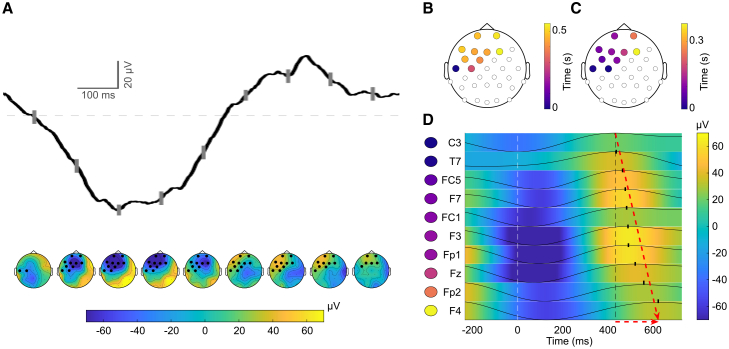
Traveling wave detection (A–C) Exemplary traveling wave with topographical plots illustrating voltage change across time. The waveform was averaged across all involved sensors (highlighted with black circles in the topographies). Gray lines mark timepoints of average voltage shown in the respective topography below. Positive voltage shown in yellow, negative in blue. The spatial trajectory of the wave is illustrated in topographical plots by varying sensor colors for the onset time of (B) troughs and (C) peaks of slow waves at each involved sensor. Purple colors denote earlier, yellow colors later onsets. (D) Averaged waveforms (filtered in the delta-band) for all sensors involved in the exemplary traveling wave, sorted by peak onset times (see C). Markers indicate peak times of each wave, and color denotes voltage amplitude (blue negative, yellow positive). Time axis is defined relative to the first trough in the traveling wave. Red arrows indicate the direction and magnitude of peak shift in time across sensors.

Given that stimulation had occurred continuously throughout the nap opportunity and significantly modulated sleep architecture and physiology, stimulation may not only have reduced the number of slow waves but also influenced their functional properties. According to theories of active systems consolidation, slow waves in N2 and SWS are crucial for memory stabilization.^9^ Slow waves are thought to modulate neural excitability while traveling along brain networks and may therefore be a vehicle to coordinate information across different neural populations.^23^^,^^26^ We identified traveling slow waves by detecting slow waves in each individual electrode during NREM stages N2 and SWS and clustering those that occurred within 200 ms in adjacent electrodes into traveling events (Figures 4A–4C*,* STAR Methods). In accordance with the stimulation-related decrease in slow waves overall, we confirmed the expected significant decrease in traveling slow waves (TSWs) across channels in the stimulation (*M* = 2526.25, *SE* = 311.80) versus the non-stimulation condition (*M* = 4870.60, *SE* = 550.46; *t*_19_ = 3.92, *p* < 0.001, *d* = 0.88). Importantly, the number of identified TSWs correlated positively with behavioral performance in the figural memory task (*r*_*rm*_ = 0.52, *p* = 0.017). While this relationship was particularly pronounced in the stimulation condition (*r* = 0.57, *p* = 0.009), it also trended in the sham condition (*r* = 0.39, *p* = 0.091). Traveling slow waves may thus be related to declarative memory consolidation.

To account for the differing sleep architecture and slow wave counts across conditions, we also analyzed TSW count normalized by slow wave count in and by the duration of established NREM, respectively (count: *t*_19_ = −1.46, *p* = 0.160, *d* = −0.33; duration: *t*_19_ = 3.73, *p* = 0.001, *d* = 0.83). No difference in TSW density between stimulation (*M* = 0.16, *SE* < 0.01) and sham conditions (*M* = 0.15, *SE* < 0.01) was observed, when normalized by slow wave count, which is perhaps unsurprising given the dependency of the clustering algorithm on detecting multiple slow waves across multiple sites: Clusters by definition consist of multiple identified slow waves that co-occur spatially and temporally. Interestingly, a differential density in TSW was retained when normalizing by NREM sleep duration, showing a higher density in the sham (*M* = 57.48, *SE* = 5.85) than in the stimulation condition (*M* = 33.61, *SE* = 4.65). As further validation of a differential density, we also calculated the TSW density in SWS only, dividing the count of TSW during SWS by the duration of SWS. In comparison to the sham condition (M = 57.48, SE = 5.85), stimulation (M = 33.61, SE = 4.65) still reduced TSW density significantly (*t*_19_ = 3.07, *p* = 0.006, *d* = 0.69), suggesting a specific decrease in TSW occurrence due to stimulation. The TSW density also significantly correlated with performance in the figural memory task overall (*r*_*rm*_ = 0.44, *p* = 0.046), and separately in both the stimulation (*r* = 0.45, *p* = 0.046) and sham conditions (*ρ* = 0.47, *p* = 0.038).

### Stimulation altered the nature and trajectory of traveling slow waves

**Figure 5:**
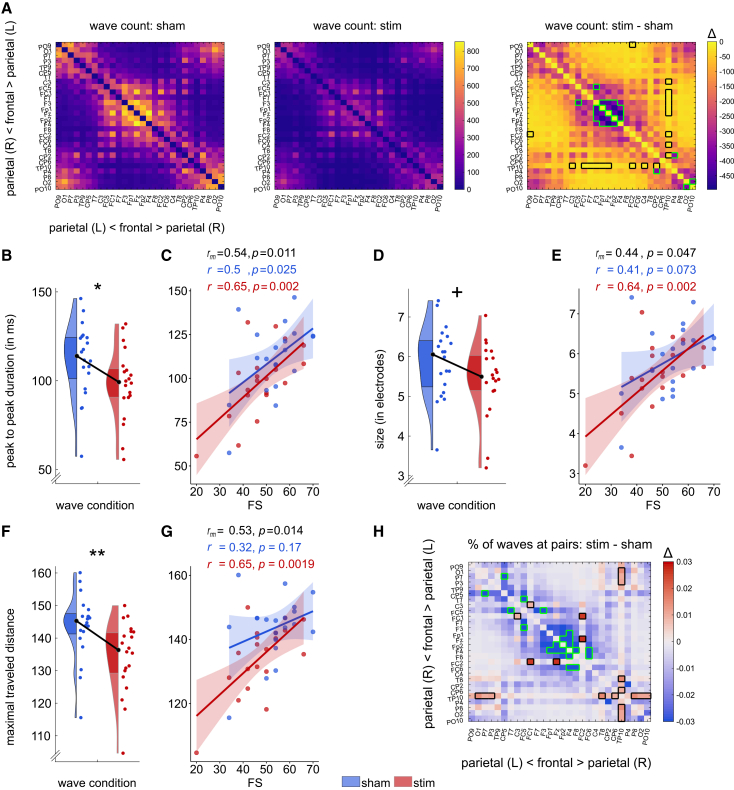
Auditory stimulation disrupts slow-wave traveling dynamics (A) Count of TSWs involving each sensor pair as a measure of cortical spread separately for sham and stimulation sessions as well as a difference map. Sensors are arranged from posterior parietal on the right via frontal to posterior parietal on the left side of the head. For the individual sessions, the yellow color indicates a higher count. For the difference, purple color indicates larger differences. Green frames indicate a significantly enhanced difference, black frames a significantly smaller difference than the average difference across conditions (p < .05). (B) Duration of wave as maximal temporal peak shift is contrasted for sham (blue) and stimulation (red) conditions. (C) Peak duration correlated positively with figural score (FS). Dots indicate individual data points, and the line represents the regression for the condition. (D) Average number of sensors involved in the TSWs is larger in the sham condition. (E) The TSW size in sensors positively correlated with the figural score. (F) Maximal distance covered by TSWs, Euclidean distance between furthest involved sensors, was significantly smaller in response to stimulation. (G) Larger traveled distance was related positively to figural score. (H) Difference in the normalized cortical spread of TSWs in each condition (count of TSWs in sensor pair divided by the overall count of TSWs in each condition) indicated broader scalp coverage for TSWs in sham (in blue) but particularly enhanced in frontal sensors (green frames, p < 0.05). Some posterior sensor pairs showed relatively more involvement for the stimulation condition (in red, significance with black frames, p < 0.05). Sensors are again arranged from right parietal over frontal to left parietal regions. Black statistics denote repeated measures correlations. Pearson’s or Spearman’s correlations were applied depending on the normality of the analyzed data (see STAR Methods). Violin plots show median values as a black dot and quartiles as a darker shaded area on the left side, as well as condition means of the individual participants as dots on the right-hand side. Statistical analyses displayed consisted of permutation shuffled matrices (A and H: see STAR Methods), paired t-tests (B, D, and F), and correlations as denoted (C, E, and G: repeated measure correlations as r_rm_; Pearson’s as r; Spearman as ρ). Permutation matrices, paired t-tests, and repeated measure correlations were performed on the total dataset of 40 naps (*n* = 20 with two sessions each), with the other correlations being performed on the individual condition (*n* = 20). Significance levels are denoted as follows: ∗∗∗ <0.001, ∗∗ <0.01, and ∗ <0.05, + < 0.1.

Consequently, we assessed whether stimulation had an influence on the traveling dynamics of the identified slow wave clusters, thus potentially altering the cortical propagation of declarative mnemonic content. We contrasted the cortical spread of TSWs across conditions by counting how many TSWs traveled between each electrode pair for the sham and stimulation condition (Figure 5A). In general, significantly less TSW spread could be observed broadly across the scalp in response to stimulation (Figure S6A), which was consistent with the higher TSW and, in general, the high slow wave count in the sham condition. When statistically contrasting the profile of these differences across sensor pairs, the observed decrease in TSWs was significantly enhanced for frontal electrodes (Figure 5A). Therefore, both the occurrence of TSWs overall and the specific decrease in TSWs in response to stimulation are particularly concentrated in frontal regions.

Stimulation also altered features of the identified TSW beyond their count and cortical spread, relating to their duration and distance covered. Peak-to-peak duration, the temporal delay between when the TSW reaches its maximum value at different electrodes, was significantly shortened for TSWs in the stimulation (*M* = 97.99 ms, *SE* = 4.48) in contrast to the sham condition (*M* = 111.50 ms, *SE* = 4.68; *t*_19_ = 2.80, *p* = 0.011, *d* = 0.63; Figure 5B). The identified TSWs showed a trend to spread to fewer sensors in the stimulation (*M* = 5.43, *SE* = 0.21) compared to the sham condition (*M* = 5.87, *SE* = 0.20; *t*_19_ = 2.00, *p* = 0.060, *d* = 0.45; Figure 5D). The spatial reach of individual TSWs was also reduced by stimulation, showing a shorter maximal distance covered between sensors (*M* = 134.30 a.u., *SE* = 2.47) in contrast to sham (*M* = 143.53 a.u., *SE* = 2.27; *t*_19_ = 3.36, *p* = 0.003, *d* = 0.75; Figure 5F). The reduction of mean travel speed by stimulation remained at the trend level (*t*_19_ = 2.03, *p* = 0.057, *d* = 0.45). The effects of auditory stimulation thus go beyond a mere reduction in the occurrence of slow waves and the time spent in SWS.

Finally, considering the spatial distortion of TSWs due to stimulation, we estimated the traveling profiles by normalizing waves engaging different electrode pairs by dividing the count of TSWs in the sensor pair by the total count of TSWs in that condition. The normalization centers the values around zero, thus controlling for condition differences in TSW count. Again, we found a significantly broader spatial spread of TSWs across the whole scalp in the sham condition (Figure S6B), suggesting that stimulation restricted TSW expanse. We contrasted the reduction values across sensor pairs, which revealed that the main cost of stimulation in terms of TSW spread was significantly focused on frontal electrodes (Figure 5H), confirming our previous results. Overall, stimulation resulted in shorter TSWs with smaller spread to fewer sensors and less spatial expanse, particularly impacting frontal regions.

### Alterations of traveling slow waves relate to declarative memory cost beyond mere SWS duration

The features of TSWs impacted by stimulation again correlated with behavioral performance. Peak-to-peak duration of the TSWs correlated positively with improved figural memory (*r*_*rm*_ = 0.54, *p* = 0.011), with the relationship holding for both sham and stimulation sessions individually (Figure 5C). Furthermore, a higher number of involved electrodes in the TSWs correlated with better memory (*r*_*rm*_ = 0.44*, p* = 0.047), significantly for stim and at a trend level for the sham condition (Figure 5E). Similarly, the distance covered by the TSWs related positively with figural memory (*r*_*rm*_ = 0.53, *p* = 0.014), even though this correlation was significant only for the stimulation condition (Figure 5G). Further, the speed with which TSWs traveled (TSW duration by maximal cortical spread) was correlated on a trend level with figural memory (*r*_*rm*_ = 0.39, *p* = 0.080). The condition-specific correlations reached significance for the stimulation session only (stimulation: ρ = 0.50, *p* = 0.026, sham: *ρ* = 0.22, *p* = 0.350). Together, this demonstrates that markers of traveling slow waves are consistently related to declarative memory performance.

### Traveling slow wave dynamics drive the sleep effect on memory

Given that both the relative time spent in SWS and TSW features correlated with behavioral performance, it remained unclear whether the effect of altered TSW features on behavior was meaningful beyond the evident effect of reduced SWS sleep, and how these effects might relate mechanistically. We answered these questions in a two-step process, by first fitting linear mixed-effects models to evaluate unique contributions of these predictors, and secondly conducting mediation analyses to explore whether the SWS benefit might be driven by TSW features, using condition as a covariate. Prior to the modeling, we used a principal component analysis to reduce dimensionality, with the resulting first principal component (PC1: TSW features) serving as a quality index of TSW features (variance explained: 92.62%). Since all TSW features loaded positively but only moderately onto the component (loadings: peak duration = 0.59, size in electrodes = 0.58, maximal distance traveled = 0.56), the component represented common qualities of the traveling wave, with each feature still retaining a substantial unique variance. Whilst we used the component “TSW features” in the main analysis to capture coordinated slow wave travel, we conducted exploratory analyses for each of the individual TSW features (see Tables S3 and S4).

#### SWS duration

Predictors showed low-to-moderate collinearity, allowing for simultaneous estimation within models (VIF: SWS = 2.08, TSW features = 1.38, condition = 2.08). According to the linear mixed-effects model, the TSW features significantly predicted memory performance even when controlling for relative time spent in SWS and condition (*β* = 0.13, *SE* = 0.05, *t*_27.1_ = 2.30, *p* = 0.0296). The stimulation condition did not predict memory performance beyond the other predictors (*β* = −0.10, *SE* = 0.20, *t*_28.1_ = −0.50, *p* = 0.619). Relative duration in SWS remained at the trend level when controlling for the other factors (*β* = 0.29, *SE* = 0.15, *t*_34.6_ = 1.97, *p* = 0.0572). Adding the TSW features significantly improved model fit over a reduced SWS-only model (χ^2^(1) = 5.21, *p* = 0.022), suggesting that TSW features provided a unique explanatory contribution. These results were further confirmed by exploratory analyses showing that the predictive contributions of the individual TSW features each improved model fit (peak duration: *p* = 0.010, size in electrodes: *p* = 0.011, maximal distance traveled: *p* = 0.037, see Table S3).

The subsequent mediation analysis confirmed that increasing relative duration in SWS came with increased memory performance (total effect: *β* = 0.59, *95% CI* = [0.284, 0.807], *p* < 0.001). Yet, it also showed that TSW features (PC1) served as a mechanistic pathway between relative time spent in SWS and memory performance. Greater relative time spent in SWS increased TSW features (path a: *β* = 0.99, *95% CI* = [0.235, 1.629], *p* = 0.004), which in turn predicted improved memory performance (path b: *β* = 0.20, *95% CI* = [0.001, 0.355], *p* = 0.032). Despite the indirect path accounting for some of the variance, it did not reach significance (path ab: *β* = 0.19, *95% CI* = [-0.010, 0.469], *p* = 0.114), with confidence intervals just crossing zero. Stimulation did not significantly alter this process (*p* = 0.536). When controlling for TSW features, the relative time spent in SWS still directly predicted memory (path c: *β* = 0.40, *95% CI* = [0.095, 0.640], *p* = 0.004). The pattern suggests that there is a predominant direct effect of relative time spent in SWS on memory performance, yet there is a mechanism by which TSW features partially mediated the effect of time in SWS on memory performance. Indeed, exploratory analyses on the individual TSW features confirmed this pattern (Table S4).

#### N2 duration

Our understanding of the relationship between sleep physiology, TSW features, and declarative memory could be strengthened by considering the relative increase in N2 duration: More N2 sleep could have given the brain less opportunity to form high-quality TSW, which might constitute the mechanism for decreased declarative memory performance. To investigate this consideration, we again performed a linear mixed-effects model and a mediation analysis, now using z-scored relative time spent in N2 as a predictor. Predictors showed low-to-moderate collinearity, allowing for simultaneous estimation (VIF: N2 = 1.98, TSW features = 1.60, condition = 1.70). In the linear mixed-effects model, relative time spent in N2 no longer had predictive value for memory, once TSW features were considered (*β* = −0.13, *SE* = 0.13, *t*_23.2_ = −1.00, *p* = 0.327). In contrast, TSW features remained weakly predictive beyond the other factors (*β* = 0.12, *SE* = 0.06, *t*_25.2_ = 1.94, *p* = 0.0642), and adding TSW features improved model fit over a reduced N2-only model at the trend level (χ^2^(1) = 3.67, *p* = 0.055). Whilst the trend-level effects need to be interpreted with caution, this pattern highlights the importance of TSW features and suggests that when TSW features are statistically controlled for, the relative time spent in N2 has no independent effect on declarative memory performance. The subsequent mediation analysis confirmed the detrimental effect of increasing time in N2 on TSW features (path a: *β* = −1.16, 95% CI = [−1.720, −0.391], *p* = 0.001). The TSW features themselves still had a beneficial effect on memory performance (path b: *β* = 0.20, 95% CI = [0.073, 0.323], *p* = 0.001). Put together, the mediation found a significant negative indirect path (path ab: *β* = −0.24, 95% CI = [−0.475, −0.040], *p* = 0.039), suggesting that the negative effect of increased relative N2 duration on memory was mediated via TSW features. Indeed, the observation that the negative direct effect of relative time in N2 on memory was not significant (path c: *β* = −0.29, 95% CI = [−0.696, 0.099], *p* = 0.167) indicated a (almost) full mediation of the N2 cost via the features of the remaining TSW.

#### Slow wave count

Given the strong effect of stimulation on slow wave count, the possibility remained that the predictive and mechanistic contributions of the TSW features might be merely epiphenomenal to the large differences in slow wave occurrence across conditions. We therefore conducted another set of a linear mixed-effect model and a mediation analysis, using z-scored slow wave count as a predictor. Predictors showed moderate collinearity, allowing for simultaneous estimation (VIF: slow wave count = 2.92, TSW features = 2.32, condition = 1.68). Both TSW features (*β* = 0.15, *SE* = 0.07, *t*_29.5_ = 2.07, *p* = 0.0476) and stimulation condition (*β* = −0.40, *SE* = 0.18, *t*_17.4_ = −2.24, *p* = 0.038) retained significant predictive value for memory when all other factors were controlled for, showing the expected positive and negative relationship, respectively. In contrast, mere slow wave count had no predictive value for memory performance, when TSW features were accounted for (*β* = −0.04, *SE* = 0.14, *t*_22.9_ = −0.31, *p* = 0.764). Therefore, while slow wave count and TSW features were moderately correlated (*r* = 0.66), only TSW features had a unique predictive value for memory. Indeed, adding TSW features significantly improved the model fit over a reduced slow wave count-only model (χ^2^(1) = 4.26, *p* = 0.0389). Moreover, the subsequent mediation analysis suggested a full mediation of the total effect of slow wave count on memory (total effect: *β* = 0.48, 95% CI = [0.103, 0.850], *p* = 0.011) via TSW features: Slow wave count significantly affected TSW features (path a: *β* = 1.38, 95% CI = [0.719, 2.059], *p* < 0.001), which in turn improve memory performance (path b: *β* = 0.23, 95% CI = [0.026, 0.394], *p* = 0.014). This mechanistic pathway was highlighted by a significant positive indirect pathway (path ab: *β* = 0.32, 95% CI = [0.029, 0.626], *p* = 0.038). However, there was no significant remaining direct path from slow wave count to memory performance (path c: *β* = 0.16, *95% CI* = [-0.169, 0.545], *p* = 0.365). These analyses indicated that the benefit of SWS was driven by the features of the TSWs rather than by their quantity.

## Discussion

Our results unveil a putative relationship between the dynamic features of slow waves and memory consolidation: Beyond the mere presence of slow waves, their traveling trajectories also actively contribute to effective declarative memory processing in sleep. We found that auditory stimulation during sleep decreased several markers of SWS quality, leading to lower delta power and fewer emerging slow waves, which were related to impaired declarative memory performance. Notably, only the figural subcomponent of the declarative memory tests was affected by stimulation and subsequently analyzed and referred to as “declarative memory,” whilst verbal memory remained intact. This suggests that visuospatial memory, in particular, may benefit from slow-wave-rich sleep, potentially due to its strong reliance on hippocampal processing. Furthermore, stimulation decreased the number of traveling slow waves, with the remaining events being restricted in duration, size, and spatial expanse. Importantly, these features of traveling slow waves were independently predictive of declarative memory consolidation, indicating that sounds presented during sleep prevented successful memory processing not only by suppressing slow wave activity, but also by hindering the natural propagation of occurring slow waves.

The observation that sounds during sleep came at a cost to sleep depth rather than overall duration replicates findings in the literature.^4^^,^^19^ Whilst duration was left marginally reduced, auditory stimulation specifically appeared to interfere with entering and maintaining SWS, instead resulting in a higher proportion of N2 sleep. This physiological alteration could be triggered by different, complementary mechanisms: The stimulation-dependent decrease in slow wave density might have prevented epochs from reaching the necessary scoring criteria of 20% slow waves. Also, auditory stimulation could trigger an arousal response, resulting in a higher likelihood of moving to lighter sleep with fewer slow waves (see below). However, differences in relative sleep duration were not driven by sub-threshold slow waves in the stimulation condition, given that slow-wave amplitudes did not differ across conditions. Importantly, the relative increase in N2 could account for the observed lack of stimulation-induced spindle reduction, given that sleep spindles occur in both SWS and N2. Taken together, our findings that auditory stimulation decreased SWS, disrupted slow waves, and impaired declarative memory consolidation support theories of active systems consolidation during sleep,^9^^,^^10^ which stress the importance of a coordinated interplay between slow waves, spindles, and ripples for declarative memory processing. The importance of slow waves for declarative memory consolidation is supported by studies emphasizing mnemonic gain or loss depending on slow wave increase^13^^,^^27^^,^^28^ or decrease.^29^

Our results further suggest that memory consolidation depends not only on the occurrence of slow waves, but also on their dynamic traveling trajectories and traveling profile across the scalp: Stimulation stunted the expanse of traveling slow waves in terms of involved sensors, duration, and physical reach across the scalp. These altered features in turn correlated with impaired memory performance, indicating that the dynamic features of traveling slow waves have a role in declarative memory consolidation. Importantly, as indicated by the linear mixed-effect models and mediation analyses, TSW features themselves predicted behavioral performance above and beyond reduced SWS sleep, and at least partially acted by mediating the impacts of altered sleep profiles and specifically the reduced slow wave count in the stimulation condition. In general, traveling waves observed across different frequency bands and spatial regions have been associated with various processes involved in computation and information propagation.^26^ For example, hippocampal theta oscillations do not just represent behavioral information but also exist as traveling waves,^30^^,^^31^ with their propagation dynamics relating to behavioral task performance.^8^ Spindles, which have been shown to travel globally across brain regions along preferred spatiotemporal paths,^32^ demonstrate highly organized neuronal co-firing depending on their propagation pattern, arguably preparing plasticity in local cortical regions.^33^ Slow waves can also travel in complex cortical patterns^23^^,^^34^ and have been associated with information transfer across brain hemispheres.^20^^,^^35^ Indeed, changes in traveling profiles of slow waves during development from childhood to adulthood related to brain-wide connectivity,^21^ while in the aging brain, tau pathology observed in diseases affecting long-term memory stunted the traveling profiles of slow waves.^24^ Interestingly, during memory tasks, different traveling patterns of waves have been associated with particular cognitive processes or mnemonic content.^36^ The functional role of traveling waves in cross-regional connectivity and (mnemonic) information transfer aligns with our interpretation that the dynamic traveling profile of sleep slow waves facilitates the propagation of information necessary for successful memory consolidation during sleep. We find that experimentally disrupting SWS, including the occurrence and traveling properties of slow waves, impairs memory performance. Given that auditory stimulation affected both the overall count as well as the characteristics of individual slow waves, we can only provide correlational evidence that both the number and expression of traveling slow waves are involved in declarative memory consolidation with shared and unique contributions. Future studies should aim at disentangling the specific contributions of the propagation of mnemonic information and slow wave generation, more generally. Potentially, time-locked stimulation^13^^,^^19^ could be implemented to disrupt slow wave activity with a specific focus on dynamic features while retaining sleep depth.

Although we observed that stimulation affected the depth of NREM sleep and traveling slow waves, which were related to declarative memory consolidation, we did not observe a detrimental effect of auditory stimulation on procedural memory consolidation. Sleep-dependent processing of procedural memories has frequently been linked to spindle activity.^12^^,^^37^^,^^38^^,^^39^ Therefore, the specific decrease in SWS should not affect the processing of procedural content, especially if compensated by an increase in N2.^40^ The dissociation between declarative cost and procedural stability corroborates findings in the literature speaking to a dissociation between the sleep-related consolidation processes of declarative and procedural memories.^3^ Plihal and Born (1997) observed a larger reliance of declarative memory processing (paired-associate lists) on SWS-heavy early night sleep, whilst procedural tasks (mirror tracing) benefited more from later night sleep, predominant in N2 and REM phases. Increasing SWS using a GABA agonist can actually deteriorate procedural memory consolidation.^40^^,^^41^ Overall, the shift from SWS to lighter sleep stages caused by the auditory stimulation appears to affect predominantly declarative memory consolidation, while sparing procedural memory processing.

One factor contributing to reduced deep sleep in the stimulation condition could be arousal following the auditory cue. Salient sensory input during sleep can evoke k-complexes, which may represent either an arousal or gating mechanism on whether the information needs to be processed and an awakening should follow.^42^^,^^43^ In this study, we observed a broad cortical response time-locked to the stimulation, indicating the triggering of k-complexes. Sound-induced arousal mediated by k-complexes might lead to a transition from slow-wave sleep to lighter sleep, such as N2.^44^^,^^45^ The proportional increase of slow-wave spindle coupling could provide further evidence for stimulation-triggered k complexes: A larger proportion of identified slow waves in the stimulation condition could be acoustically evoked k-complexes, which have been linked to subsequent spindles,^46^ thereby increasing the relative co-occurrence of slow waves and spindles. Interestingly, some authors have also made a distinction between type I (k complex-like) and type II (homeostatically regulated) slow waves^47^: The auditory stimulation in the current study may have triggered type I slow waves and decreased the occurrence of type II slow waves. Our analyses of the traveling wave forms support this idea: On average, the detected traveling waves exhibited a shorter duration for the stimulation condition, similar to the difference observed between type I and type II waves. Type I waves are also associated with an increase in high-frequency activity, which could be observed in the stimulation condition. Subsequently, power in the lower frequency bands, encompassing delta, theta, and spindle bands, was suppressed, which is coherent with the previously reported type I wave-bound suppression of the spindle frequency band.^47^ Importantly, spindle analyses provide further support for the arousal hypothesis, by showing that stimulation caused an increase in peak spindle frequency for fast spindles. Previous research has linked the speed of peak spindle frequencies to sleep depth, indicating faster spindles during nap periods compared to night sleep^48^ and during lighter sleep versus deep sleep.^49^^,^^50^ An increase in peak frequency of fast spindles for auditory stimulation could thus be an indicator of lighter sleep or arousal. The stimulation-related increase in peak fast spindle frequency is also consistent with the observation that externally elicited spindles are faster than spontaneously occurring ones.^51^ In line with the present study, when k complexes interrupted concurrent spindles, subsequent spindles were found to increase in frequency by about 1 Hz.^52^ Interestingly, an increase in fast spindles was observed in response to auditory TMR^11^ and to auditory stimulation in-phase with slow waves.^13^ Thus, auditory stimulation during sleep may cause arousal responses even if they are timed to the slow wave phase or act as memory reactivation cues.

The arousal(-like) response to auditory stimulation during sleep contrasts with the success of TMR and closed-loop stimulation of slow waves in promoting memory consolidation and thus raises both functional and methodological questions. Firstly, given the ostensible success of auditory TMR to boost memory retention,^16^ it could even be possible that content-loaded stimuli are processed differently to random noises, or that the gain derived from triggering a reactivation event outweighs the arousal costs, leading to a net benefit. The increased response strength in the spindle frequency band evoked by memory-associated cues compared to neutral cues supports the idea that post-cue processing depends on stimulus content.^11^ Further studies are needed that explicitly manipulate the balance of costs and benefits for presenting content-loaded auditory stimuli during sleep. Secondly, the specific impact of the auditory stimulation is likely bound by its temporal relation to ongoing slow wave activity. Closed-loop algorithms stimulating during the up-phase of the slow wave have been shown to enhance slow wave activity.^13^^,^^18^^,^^44^ Importantly, Ngo and colleagues (2013) observed that the traveling profiles of spontaneously occurring and induced slow waves, i.e., stimulated when the next predicted slow wave up-phase should occur, were comparable across a range of measures. Combined with our finding of randomly timed stimulation disturbing both slow wave activity and their usual traveling profiles, it appears that the precision of the algorithm timing may underlie this advantage of closed-loop stimulation. Indeed, using detection algorithms to stimulate slow waves out of phase can successfully disrupt slow-wave activity and thereby suppress slow-wave sleep in humans.^19^ Interestingly, this closed-loop suppression produced a comparable reduction in SWS to the random auditory stimulation performed in this study. Therefore, at least for SWS suppression, random auditory stimulation might be a suitable, simplified alternative to phase-locked stimulation. Developing a simple and reliable method to disrupt slow-wave sleep has clinical and experimental value, such as exploring the role of SWS in emotional memory processing or modeling sleep fragmentation in different neurological disorders. The negative effects of random noise on sleep and cognition are also relevant to society, as sleep duration alone is not a valuable predictor for sleep health; also, sleep depth and the properties of sleep oscillatory activity need to be considered. Taken together, the detrimental impact of acoustic stimulation on slow waves and their profiles that we observed might contextualize null findings across the literature, for which stimulation did not improve memory consolidation^53^^,^^54^: Potential benefits of stimulation, whether content-loaded or timed accurately, may have been outweighed by the cost of sounds during sleep.

### Limitations of the study

The perceived intensity of auditory stimulation in this study was difficult to measure audiometrically, given the use of insert earphones. While it was possible to ensure that the volume of auditory stimulation exceeded their individual hearing thresholds, the reported decibel levels of the said hearing threshold and subsequently the stimulation volume were merely estimations based on biological normalization. As a consequence, the volume of stimulation was possibly greater in this study than in usual TMR paradigms, increasing the chance of sleep disturbances. Whilst we did not observe such stimulation-driven sleep disturbances (as measured by wake post sleep onset), the existing differences in auditory stimulation to TMR need to be considered when drawing general conclusions from our findings regarding the presentation of auditory stimuli during sleep: It is possible that the increased volume was disproportionally affecting sleep and memory outcomes in the current study in a way that lower TMR volumes would not, thereby giving way to TMR-related memory improvement. Indeed, home applications of TMR have proven unreliable, partially due to the difficulty in adjusting to the right volume^55^ and studies have shown TMR to be detrimental when the noise is disruptive.^56^ A subsequent study with confirmed equivalent sound levels to TMR studies would be required to differentiate whether sounds disturb traveling profiles in addition to reactivating memories in these settings, and to delineate the effect of this potential disturbance on memory consolidation. Further, in the current study, the sounds were presented prior to sleep onset, which marks an important deviation from many TMR studies since participants were aware of the sounds being presented. It is possible that this led to an active suppression effort by the participants, and it cannot be ruled out that some remaining auditory suppression lingered during sleep. Despite the current sounds not being content-loaded, as is usual in TMR settings, the conscious presentation of sounds could have had unintended downstream effects that only a further study with random sounds presented exclusively after sleep onset could disentangle. Additionally, it should be noted that only male subjects were tested in this study and that the sample size was restricted. There was no expectation of sex-related differences in the discussed sleep and memory mechanisms, yet they cannot be excluded based on this sample. Further studies with a more varied sample and a larger sample size would be needed to corroborate our results.

In conclusion, our results critically extend existing literature on auditory stimulation in sleep by showing that random stimulation disturbs specifically SWS, slow wave occurrence, and their traveling profiles, at the cost of declarative memory consolidation. Whilst supporting current theories of sleep-dependent memory consolidation, our findings highlight the need to consider dynamic features of sleep physiology beyond average markers like sleep duration. Furthermore, the observation that stimulation tied to learning content or not timed to ongoing oscillatory activity decreases memory performance has methodological implications for the use of targeted memory reactivation and time-locked stimulation paradigms.

## Resource availability

### Lead contact

Requests for further information and resources should be directed to and will be fulfilled by the lead contact, Nora M. Roüast (nora.roueast@psychologie.uni-freiburg.de).

### Materials availability

This study did not generate new unique materials.

### Data and code availability

• All data necessary to replicate the findings reported in this paper have been deposited at the Open Science Framework: https://doi.org/10.17605/OSF.IO/WJZS9 and FreiData: https://doi.org/10.60493/8t6p9-xfq49.
• Transcribed behavioral data reported in this paper have been deposited at the Open Science Framework: https://doi.org/10.17605/OSF.IO/WJZS9.
• All original code has been deposited at the Open Science Framework: https://doi.org/10.17605/OSF.IO/WJZS9.
• Any additional information required to reanalyze the data reported in this paper is available from the lead contact upon request.

## Acknowledgments

We thank Susanne Möhrle for help with data acquisition and Angelina Eisele for her contribution to data visualization. This work was supported by 10.13039/501100002347BMBF funding and a 10.13039/501100001659German Research Foundation Emmy Noether grant to Monika Schönauer and Steffen Gais (SCHO1820/2–1, GA730/3-1), and a 10.13039/501100001659German Research Foundation Walter Benjamin grant to Nora M. Roüast (RO6828/1-1, RO6828/1–2).

## Author contributions

S.G. and M.S. contributed to the design and implementation of the research. N.M.R. completed data preparation, statistical analyses, and writing of the manuscript. D.K. completed the phase coupling analysis and provided feedback on the manuscript. M.S. further contributed to the writing of the manuscript. S.G. also provided feedback on the manuscript.

## Declaration of interests

The authors declare no competing interests.

## STAR★Methods

### Key resources table

**Table:** 

REAGENT or RESOURCE	SOURCE	IDENTIFIER
**Deposited data**		

EEG data	–	FreiData: https://doi.org/10.60493/8t6p9-xfq49
behavior data	–	OSF: https://doi.org/10.17605/OSF.IO/WJZS9
custom code	–	OSF: https://doi.org/10.17605/OSF.IO/WJZS9

**Software and algorithms**		

MATLAB R2020b	Mathworks	https://www.mathworks.com/; RRID: SCR_001622
FieldTrip Version: 20230418	Oostenveld et al.^57^	RRID: SCR_004849
Violin plots for MATLAB	Bechtold^58^	https://doi.org/10.5281/zenodo.4559847
RStudio 2023.12.1 + 420	R Studio Team	RRID: SCR_000432
R 4.5.3 (2026-03-11)	R Core Development Team	https://www.r-project.org/; RRID: SCR_001905
lme4	R Project for Statistical Computing	https://www.r-project.org/; RRID: SCR_015654
repeated measures correlation (rmcorr)	Bakdash and Marusich^59^	https://doi.org/10.3389/fpsyg.2017.00456
lavaan	Rosseel^60^	https://doi.org/10.18637/jss.v048.i02
Lern-und-Gedächtnistest, LGT-3	Bäumler^61^	Bäumler^61^
EEGLAB version 2023.0	Delorme and Makeig^62^	https://eeglab.org/; RRID: SCR_007292

**Other**		

Brain Products	EEG System	https://www.brainproducts.com/; RRID: SCR_009443

### Experimental model and study participant details

Twenty-five healthy male and native German-speaking adults were recruited for this study. At the time of data collection, evidence suggested that the female cycle might have an effect on sleep-related memory consolidation.^63^ To preclude potentially confounding effects, we admitted only male participants to this study.

All participants were non-smokers, right-handed (as assessed by the Edinburgh Handedness Scale^64^), and had no diagnosed sleep- or memory-disorders. None consumed drugs or were prescribed medication that could affect their central nervous system. The participants had a regular sleep rhythm of 7–9 h (measured by the Munich Chronotype Questionnaire^65^ and did not work in shifts or experienced jet lag in the last six weeks before the study took place. Further task-specific exclusion criteria were applied: Piano players and Turkish speakers were excluded to ensure measure efficacy in the finger tapping and LGT-3 tasks, respectively. Distinct and superior finger dexterity in piano players^66^ would bias performance, given that finger tapping is similar to piano playing. The LGT-3 includes learning vocabulary in a foreign language (Turkish), which would become redundant if the language was already known.

Five participants had to be excluded due to withdrawing from the study prior to the second session (*n* = 2), equipment failure in recording electrophysiology continuously (*n* = 2) or due to a failure to fall asleep in an experimental session, defined as at least 20 min in sleep stage N2 or deeper (*n* = 1). All analyses were performed on the data of the remaining set of twenty participants (age: *M* = 24.05, *SD* = 3.91, range: 18–31).

The experiments were approved by the ethics committee of the Department of Psychology, LMU Munich (reference GA730/3-1, 18th Jul 2009). Informed consent was obtained from all participants. Data collection for this project took place from May through August 2011.

### Method details

#### Procedure

The study was carried out on two experimental visits, separated by at least six days (*M* = 17.95, *SD* = 12.17), each including declarative and procedural memory tasks as well as a 3-h sleep period (nap). The task versions and the order of the stimulation conditions during the nap were randomized and counterbalanced across participants (Figure 1A). Participants were recompensed for their participation at a rate of 8 Euros per hour.

In preparation for the study, participants completed the Munich Chronotype Questionnaire^65^ to evaluate regular sleeping behavior. In order to increase the likelihood of sleep during the nap opportunity, participants were asked to go to bed regularly the three preceding nights but rise an hour earlier than usual. Additionally, participants were asked to refrain from consuming caffeine or medication on the experimental days as well as alcohol on the days of and preceding the experiment. A hearing test was administered to assess participants’ hearing threshold prior to the experiment. The encoding session was equivalent for both experimental visits, consisting of one version of the LGT-3, followed by one type of sequence learned in the finger tapping task.

Participants then had a 3-h nap opportunity, during which EEG was recorded. In the stimulation condition, a train of six clicks (10 Hz stimulation train, overall lasting 600 ms) was presented every 9.5 s (*M* = 9.5313, *SD* = 0.0013) via insert earphones (see Hearing test and auditory stimulation). No sounds were presented during the sham condition. After the nap opportunity, participants completed the retrieval phase, consisting of the recall of the declarative information learned in the LGT-3 set and the performance of the finger tapping task. At the end of each experimental day, a psychomotor vigilance task was performed.

#### Task material

##### Hearing test and auditory stimulation

Within the first session, hearing thresholds were tested to ensure that subsequent auditory stimulation would exceed the individual participant’s hearing threshold. Via MATLAB, a continuous sinus tone with a frequency of 4000 Hz was presented via insert earphones, which steadily increased or decreased in volume. Participants had to press the “up” or “down” key respectively, if they could hear it for the first time (getting louder) or for the last time (getting quieter). Increase and decrease trials were sampled in random order. Hearing threshold was assessed as the mean individual measured output source values, recalculated as decibel level (*M* = 15.50 dB SPL, *SE* = 2.39). Auditory stimulation consisted of a train of 6 clicks at 10 Hz, repeated around every 9.5 s (*M* = 9.53 s, *SD* < 0.01). Prior to the nap, participants adjusted the stimulation volume themselves from a set starting sound level (60.92 dB SPL) according to the instruction to keep sound levels as high as possible, but low enough to fall asleep to. Experimenters ensured that the chosen level was above their individual hearing threshold, resulting in an average stimulation intensity of 65.26 dB SPL (*SE* = 0.75), which is comparable to the seminal paper on auditory stimulation during sleep (∼62 dB SPL^67^). Auditory stimulation was presented using insert earphones and throughout the nap period, including during falling asleep and waking up.

Since we did not obtain valid audiometric measurements of the stimulation sound pressure per participant, we estimated the stated sound pressure levels (dB SPL) based on (1) the original recorded digital amplitude units for both hearing threshold (ht) and stimulation volume, (2) the standard physical computation for decibel (*htdB*_*SPL*_ = 20∗*log*_10_(*ht*)), and (3) the biological norm sound pressure levels for hearing thresholds using insert headphones at 4000 Hz (namely, 15.5 dB SPL according to ISO 389-2^68^). The average measured hearing threshold across participants was aligned with the norm hearing threshold for such a setup (ISO 389-2), resulting in an offset of 76.81 dB SPL. This offset was applied to both hearing threshold and stimulation volume to extrapolate biologically plausible stimulation volumes. It should be stressed that these measures were computationally derived using standard norms and not measured with a sound meter. Further, it should be noted that the use of insert earphones led to higher threshold and stimulation volumes, with the international standard showing a 6 dB increase in hearing thresholds for insert earphones in comparison to supra-aural headphones (9.5 dB SPL, ISO 389-1^69^). Correcting for the use of insert earphones, we estimated a relative sound level of stimulation above their hearing threshold of around 50 dB SL (*M* = 49.76 dB SL, *SE* = 2.26), which is slightly louder than the perceptual intensity levels reported by some in the literature (e.g., 40 dB SL,^11^ 50 dB SPL using speakers^7^). An increase in sound level for the stimulation was particularly relevant in this study given the differing auditive properties of the stimuli used: The clicks for stimulation required higher sound intensity than the tone assessing hearing thresholds, given that their lower frequency (<500 Hz^70^) corresponded to decreased spectral sensitivity.

##### Learning-and-memory-task (Lern-und-Gedächtnistest, LGT-3)

The LGT-3 is a German test battery of different paper-and-pencil declarative long-term memory tasks,^61^ assessing a range of declarative skills (e.g., figural or verbal) with different retrieval types (e.g., free recall or recognition) to access different aspects of declarative memory. Two equally difficult versions of the test battery exist, allowing to contrast condition effects across sessions. Each of these versions consist of six distinct subtests, named “city map”, “signs”, “vocabulary”, “phone numbers”, “objects”, and “story details”, which are presented in a standardized, fixed order during the encoding and retrieval phases. During encoding of “city map”, subjects had to memorize a path through a complex maze connecting a start and end position within 1 min; whilst during retrieval they were given 2 min to redraw this path. The encoding of “signs” consisted of remembering made-up brand logos, i.e., abstract and concrete drawing within distinct frames, within 1 min; during retrieval the logos had to be matched to a choice of four frames within 4 min “Vocabulary” tasked subjects with learning 20 Turkish-German word pairs within 1 min; during retrieval the German words had to be matched with the recognized Turkish translation given five choices within 4 min. During the encoding of “phone numbers” subjects associated names to 13 three-digit numbers within 2 min; during retrieval they were cued with the name and had to recall the associated three digits within 2 min. Twenty simple “objects” were encoded within 1 min and freely recalled within 2 min “Story details” were encoded by reading a story within 1 min, and retrieved by answering 14 questions concerning content (e.g., names or numbers) within 4 min. The resulting values can be calculated using weighted sums to assess figural memory (figural score, FS, comprised of “city map” and “signs”), verbal memory (verbal score, VS, comprised of “vocabulary”, “phone numbers”, and “objects”) and a total declarative memory score (DMS, comprised of all elements). In this study, the two equivalently difficult task sets were randomly allocated across stimulation and sham condition. The test procedure was adapted to accommodate the 3-h nap opportunity between encoding and retrieval phase. Prior studies suggested that this delay did not hamper the measurement efficacy, and that task performance benefits from sleep in contrast to a wake condition.^4^

##### Finger tapping

Procedural learning was assessed via a finger tapping task, which is sleep-dependent.^71^^,^^72^ The motor task consisted of a sequence of five numbers that participants had to tap on a keyboard in the right order. There were two equivalently difficult versions of the sequence (FT1: “4-1-3-2-4”, FT2: “4-2-3-1-4”),^71^ and each study day was randomly assigned one version. In a fixed task window (“block”) of 30 s, participants were tasked to type the chosen sequence correctly and as often as possible, using their non-dominant hand (i.e., left in this study). For this, participants were instructed to focus on both speed and accuracy. Outcome measures per block are mean time until button presses (reaction times, “RT”, in ms), the total number of typed sequences (“count”), as well as the number of correctly typed sequences (“accuracy”). The encoding phase consisted of 12 blocks, separated by 20-second-long recovery breaks. The last three blocks of the encoding phase are used for statistical assessment, to match the three blocks implemented as the retrieval phase.

The finger tapping task was computer based with a generic keyboard and presented via MATLAB. The participants were in a darkened room and focused on the screen displaying the sequence in white script on black background. After typing a number, a star symbol (∗) appeared, indicating the current position within the sequence to the participants. Auditory feedback was provided via a speaker for wrong key presses. The participants also received an auditory alert paired with a visual prompt to indicate the start of the next block after each recovery break.

##### Psychomotor vigilance task (PVT)

This simple visual reaction time test^73^^,^^74^ was implemented to monitor alertness and vigilance for each experimental session. Four red numbers were presented on a darkened computer screen and started counting upwards in milliseconds at irregular time intervals. Participants were instructed to press the spacebar to stop the counting as quickly as possible with their dominant (right) hand as soon as they saw the numbers changing. The resulting number on the screen represented their reaction time in milliseconds. Button presses <200 ms counted as wrong (impulsive) presses, and presses >500 ms as lapses. The wrong types of presses were analyzed separately from the rest.

##### Electrophysiology recordings and preprocessing

Electroencephalography (EEG) was measured with a standard setup (10-20-System^75^) and amplifier by Brain Products, using Ag-AgCl-electrodes in the ActiCap 128 electrode system. The EEG recording was measured with 32 channels and a sampling rate of 1000Hz during the sleep period. Separate derivations for EEG, electrooculogram (EOG) and electromyogram (EMG) derivations of the complete data were created^62^ and scored in 30-s windows according to Rechtschaffen and Kales.^76^ Data preprocessing was completed using Fieldtrip (Version: 20230418,^57^ initially demeaning, detrending and filtering the data (low-pass: 140 Hz, high-pass: 0.1 Hz, notch: 50, 100, 150 Hz) each channel. Five-second trials were created locked to stimulation times or equivalent time stamps for the non-stimulation condition and used for artifact rejection: After a manual rejection of channels and trials based on variance, EOG, jump, and motion artifacts were detected based on adjusted thresholds and cut from the data. Missing channels were interpolated using the spline method before the cleaned data were then re-referenced to the average reference. Due to the data segmentation into trials for artifact rejection, the temporally continuous data were split into stimulus-locked and secondary “consecutive” segments, which were subsequently processed for time-frequency and event-related potential analyses, evaluated statistically and plotted separately but equivalently (Figures 1B and 1C).

### Quantification and statistical analysis

#### Frequency and time-frequency analyses

Frequency and time-frequency analyses of overall and stimulation-locked activity were performed using Fieldtrip. To achieve higher frequency accuracy especially for lower frequency, both analyses were completed separately. Frequency was estimated by using the entire data implementing a Hanning taper for the frequency band of 0.5–30 Hz. Statistical contrasts across conditions were calculated using cluster-based permutation testing across all frequencies, and specifically for delta and spindle frequencies (averaged across frequency band). Time-frequency decomposition of the stimulation-locked data (-1s–4s, relative to stimulation) was achieved using a Fourier analysis based on sliding time windows in steps of 50 ms, also implementing a Hanning taper. The window length was set to three cycles of the given frequency (1–30 Hz), moving in 1 Hz steps. Statistical analysis was also performed using cluster-based statistics, either across all frequencies and time-points post stimulation, or for frequencies of interest (averaging across spindle or delta band). All Fieldtrip cluster-based permutation tests (also for the following analyses) are inherently corrected for multiple comparisons.

#### Event-related potential analyses

Using Fieldtrip, the trials time-locked to stimulation times (-1s–4s, relative to stimulation) were detrended and a baseline was applied for 100 ms before stimulation onset. Event-related potentials were then calculated, averaged across trials, and statistically evaluated with cluster-based permutation testing.

#### Spindle peak-frequency

The power spectral density (PSD) was estimated using the MATLAB pwelch function, restricted to frequency range of 0.1–45 Hz. Fast Fourier Transform was computed on the signal divided into overlapping segments (by 95%) with window length of twice the sample length. The average, log-transformed PSD was then used to find peaks in the fast (13-17Hz) and slow (11-13Hz) spindle range. In accordance with literature,^49^ fast spindles were defined as beyond 13 Hz and have been associated with posterior topography.^77^

#### Spindle and slow wave detection

Cleaned trials were concatenated to create a pseudo-continuous artifact-free file, before being bandpass filtered to either spindle (11–17 Hz) or slow wave (0.5–4 Hz) frequencies. Detection algorithms were processed for each channel. The spindle detection algorithm creates an envelope around the spindle-filtered data, and identifies peaks in the envelope (minimum distance: 25 ms). The amplitude criterion was defined as exceeding the 95th percentile relative to REM peaks if present, otherwise on N1 peaks. Spindles were identified if exceeding the amplitude criterion for 0.5 s, and duplicates were deleted in favor of the maximal option. Parts of spindle events crossing non-continuous trial boundaries were excluded, unless the spindle would then not fulfill the duration criterion, in which case the whole spindle was excluded. The slow-wave detection defined peaks and troughs as well as inflection points. Identified slow waves were excluded if they crossed non-continuous trial boundaries or were below the amplitude criterion, defined as the 99th percentile of N1 sleep peaks. Slow wave or spindle density was defined as the average count of the sleep features within a channel in the stated sleep stages, divided by the duration of those respective sleep stages in minutes. Established NREM sleep refers to N2, N3, and N4.

Phase coupling analysis was conducted by extracting phase information from the inflection points, peaks, and troughs of each identified slow wave for each individual. Spindle events were assigned to a slow wave if their onset occurred within the temporal bounds of that individual slow wave for each participant and condition. The phase values of SO–spindle onset pairs were then averaged across predefined regions of interest (ROIs): frontal (F3, Fz, F4) and parietal (P3, Pz, P4). To assess the differences in phases between stimulated and sham conditions in either parietal or frontal ROIs, we used circular statistics (Hotelling’s paired sample test). In addition to the phase relationship between SOs and spindles, we examined whether the incidence of coupling was affected by stimulation. Specifically, for each slow wave cycle, we determined whether at least one spindle onset occurred within that cycle. We then calculated two complementary metrics for each ROI: (1) the proportion of slow waves containing spindles relative to total slow waves detected, computed by averaging proportions across the three channels within each ROI, and (2) the absolute count of slow waves with spindles, computed by summing counts across the abovementioned three channels within each ROI. For the proportions, we averaged the values across channels. In contrast, absolute counts were summed to capture the total magnitude of coupling events across the three channels, representing the ROI.

#### Slow wave clustering

Identified slow waves across channels were sorted by peak time. Using the fieldtrip neighborhood structure method triangulation, we identified relevant neighboring sensors for each EEG sensor, and grouped individual slow waves in clusters if peaks fell both within neighboring structures and within 200 ms of each other.^22^ Only clusters containing at least two sensors were included. Using surface EEG, the traveling trajectory of the slow wave can be observed by plotting scalp topographies at various points of the average waveform, illustrating the progressive voltage shift across sensors and time (Figure S4A). The spatial progression of the wave across sensor space is illustrated more clearly by coloring the wave troughs (Figure S4B) and peaks (Figure S4C) by onset time. The temporal shift of negative and positive peaks becomes evident when contrasting the waveforms and voltages for each sensor, demonstrating both the cumulative voltage change in sensors as well as the temporal shift of peak to peak across individual waveforms (Figure S4D).

#### Wave parameters and statistical analysis

TSW density was calculated in parallel to slow wave and spindle density above by dividing the count within the stated sleep stages by the duration within those sleep stages. A separate TSW density measure divided the count of TSWs by the count of slow waves more generally. Overall wave duration measured the difference between the first and last instance of any slow wave within the cluster, using the downward inflection points before and after trough and peak, respectively. Peak duration represents the maximum temporal shift of peaks within the clusters, thus the difference between first and last maximum of individual slow waves. The size of the cluster was defined by how many slow waves were grouped within the cluster. The maximum distance covered was calculated by the Euclidean distance between the two sensors in the cluster farthest apart. Speed was estimated by the quotient of maximal distance and total wave duration. Statistical analyses were performed in R, using t-tests or Wilcoxon Signed-Rank tests, depending on applicability of assumptions. The spread of the wave was estimated in two ways: The first measure was a simple count of waves present for each electrode pair to demonstrate where in the sensor space TSWs occurred and to put this in relation across conditions. The second measure normalized the count distribution by the total count of TSWs in the respective condition, thereby giving a distribution around zero showing where TSWs predominantly occurred irrespective of total occurrence. This second measure allowed a contrast between conditions without a distorting influence of differing slow wave count. A difference measure was calculated for both measures by subtracting the mean values across subjects in the sham from the mean values in the stimulation condition. All “real” values and following permutation values were organized in vector matrices, with each number indexing one unique sensor-on-sensor pair (i.e., below the diagonal in a heatmap). The two difference measures were statistically analyzed with two permutation approaches by either (a) shuffling condition labels within-subject or (b) shuffling vector order across subjects. For the first analysis (a), 1000 permutation difference arrays were created by looping over each subject and randomly reassigning the condition label (“flipping”) or leaving it as is, calculating the difference measure (stimulation-sham), and then averaging across subjects. This approach highlights whether and where there are differences across conditions. In the second analysis, 1000 permutation difference arrays were created by randomly rearranging the vector order of the real difference vector: shuffling the index creates random value distribution for each sensor pair by assigning the real values to a random location. Since the difference values are contrasted with just other difference values, this approach highlights where existing condition differences were particularly prominent. For both options, each actual value was deemed significant at an alpha of 0.05, if it was within the 2.5% extreme tail ends of the randomly permuted distribution of 1000 values for that sensor pair. Significance is portrayed mirrored for both representations of each sensor pair.

#### Linear mixed-effect modeling and mediation analysis

To reduce dimensionality and capture the shared variance among the TSW features, we performed a principal component analysis (PCA) on the z-scored feature values (peak duration, size in sensors, and maximal distance covered). The first principal component (PC1) explained 92.62% of the total variance and was retained for further modeling as index of TSW features. Given that all features loaded positively but only moderately on PC1 (range: 0.56 - 0.59), we ran following models on the PC1 but report separate models for each feature in the supplementary material. Subsequently, we fitted linear mixed-effects models (LME) using the lme4 package in R to assess whether TSW features added further predictive value to the figural memory score beyond the percentage in SWS or N2. The full model included fixed effects for percentage in sleep, the TSW features (PC1, or individual TSW features in supplementary material), and experimental condition (sham vs. stimulation), with a random intercept for participants to account for repeated measures. All measures were z-scored. Collinearity was assessed using variance inflation factors (VIF), confirming low-to-moderate multicollinearity (all VIF <2.5). Model fits were compared against a reduced model containing only the percentage in sleep stage and condition using likelihood ratio tests (ANOVA). Lastly, to test whether the relationship between percentage in sleep stage and memory was mediated by the TSW features, we conducted a mediation analysis using structural equation modeling (SEM) with the lavaan package in R. The model specified percentage in sleep stage as predictor, TSW features as mediator, and figural score as outcome. We further included stimulation condition as covariate to control for session-specific effects. Standard errors were estimated using cluster-robust bootstrap resampling (5000 samples) clustered by participants in order to account for the repeated measures design. Indirect effects were quantified as the product of the path from percentage in sleep stage to TSW features (path a) and the path from TSW features to figural score (path b). We further performed a parallel linear mixed-effects model and a mediation analysis using slow wave count instead of percentage in sleep stages to assess TSW features held predictive value beyond stimulation-bound differences in slow wave count. Again, all measures were z-scored and collinearity was moderate (all VIF <3).

#### Statistical analysis

Unless otherwise specified, statistical analyses were performed in R, using t-tests or Wilcoxon Signed-Rank tests as appropriate with effect sizes calculated as Cohen’s d or Wilcoxon effect size r, respectively, and plotted with violin plots.^58^ Relationships between sleep parameters and behavioral measures were assessed using Pearson or Spearman correlations, depending on the normality of the underlying data distributions. Repeated measures correlations (‘rmcorr’^59^; signified as *r*_*rm*_) were used to assess relationships of variables across conditions, thereby containing multiple measures per participant. The mediation analyses were performed using Lavaan^60^ in R. Significance levels are denoted as follows: ∗∗∗ <0.001, ∗∗ <0.01, ∗ <0.05, + < 0.1.
