# Supplementary material for: Random auditory stimulation during sleep disturbs traveling slow waves and declarative memory
Source: iScience. 2026 Jun 25;29(7):116601. doi: 10.1016/j.isci.2026.116601 (PMC13378391; doi:10.1016/j.isci.2026.116601)
Supplement: Document S1. Figures S1–S6 and Tables S1–S4 [file mmc1.pdf]

iScience, Volume *29*

Supplemental information

Random auditory stimulation during sleep disturbs traveling slow waves and declarative memory

Nora M. Roüast, Deniz Kumral, Steffen Gais, and Monika Schönauer

SUPPLEMENTAL TEXT AND FIGURES:

Supplementary Table S1. Sleep parameters for stimulation and sham condition. Duration is shown in minutes, by mean and standard error of the mean.

	sham	stimulation	difference in sleep duration
**Overall**	130.5 ± 8.7	113.1 ± 10.8	total: t_{19} = 1.91, p = .071, d = .43
**N1**	14.2 ± 2.2	13.1 ± 2.3	relative: W_{19} = 110, p = .869, r = .04
**N2**	65.1 ± 5.8	69.0 ± 6.5	relative: W_{19} = 200, p < .001, r = .79
**SWS**	25.5 ± 2.9	10.3 ± 2.4	relative: t_{19} = 5.95, p < .001, d = 1.33
**REM**	21.9 ± 4.6	20.1 ± 4.2	relative: t_{19} = .11, p = .916, d = .02
**latency**	10.7 ± 2.3	12.6 ± 4.4	total: W_{19} = 110.5, p = .852, r = .05
**wake**	20.9 ± 5.9	24.4 ± 7.3	total: t_{19} = .11, p = .916, d = .02

Supplementary Table S2. Sleep parameters across sessions. Duration is shown in minutes, by mean and standard error of the mean.

	session 1	session 2	difference in sleep duration
**Overall**	113.9 ± 10.0	129.7 ± 9.7	total: t_{19} = -1.71, p = .103, d = -.38
**N1**	12.9 ± 2.0	14.4 ± 2.5	relative: t_{19} = .22, p = .829, d = .05
**N2**	62.7 ± 6.1	71.5 ± 6.1	relative: W_{19} = 89, p = .571, d = .13
**SWS**	18.4 ± 2.9	17.4 ± 3.3	relative: t_{19} = .85, p = .406, d = .19
**REM**	19.3 ± 4.3	22.6 ± 4.6	relative: t_{19} = -.41, p = .683, d = -.09
**latency**	9.5 ± 1.9	13.9 ± 4.5	total: W_{19} = 93.5, p = .681, r = .10
**wake**	22.9 ± 6.9	22.4 ± 6.5	total: W_{19} = 80.5, p = .573, r = .13

Supplementary Figure S1. Auditory evoked responses in NREM and REM sleep. While the discussed ERP effects and individual participant data indicated that auditory stimulation triggered k complexes, not all participants showed them to the same extent or at all: Eleven participants showed immediate evoked responses, seven participants showed no evoked potentials, and two participants showed a delayed reaction. The amplitude of the k complex-like potentials appeared larger in NREM sleep stages (N2 and SWS) in contrast to REM. **(A)** Event-related potentials for NREM sleep (N2 and SWS) in response to stimulation plotted for three individual exemplary participants, one each for immediate (n=11), no (n=7), and delayed evoked potentials (n=2). ERPs showed k complex-like inflections in the stimulation condition (red) in contrast to the sham condition (blue). Lines denote trial averages, shaded area standard error. **(B)** Average ERP for NREM sleep showed significant stimulus-evoked potentials (p = .012, n=20). Red bar denotes time of significant difference across conditions. **(C)** ERPs for REM sleep in response to stimulation plotted for three example participants. **(D)** Average ERP for REM sleep in contrast showed no significant stimulus-evoked potentials (p = .239, n=11). It should be noted that only eleven participants had REM sleep in both nap sessions and could thus be statistically compared. Lines and shaded area denote mean ± SEM.

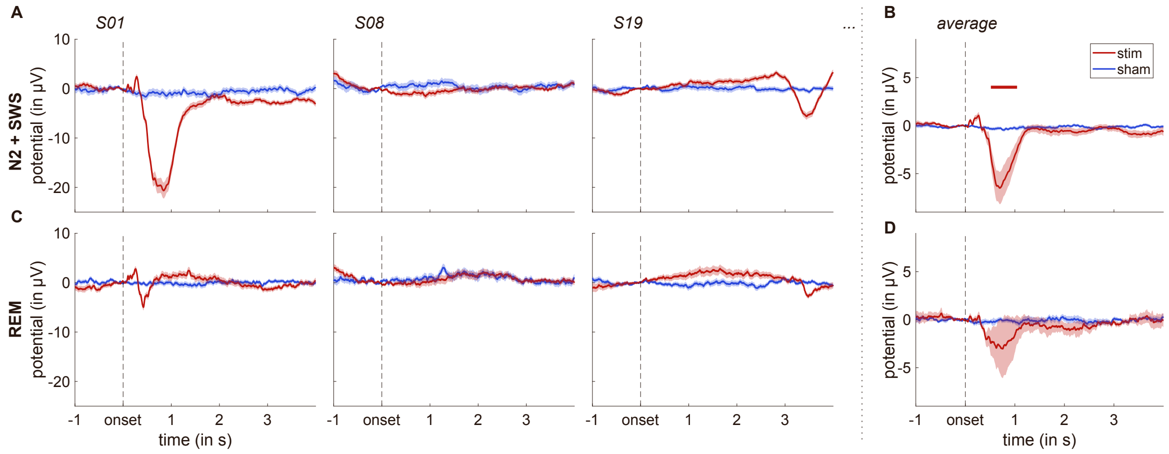

Supplementary Figure S2. Effects of auditory stimulation in participants with and without auditory evoked responses in electrical brain activity. Whilst the effects on sleep architecture and declarative memory appear to be more pronounced in participants reacting to stimulation with an evoked response (n=13), stimulation also had a similar effect in participants without such response (n=7). **(A)** Sleep duration (in seconds) in sham (shown in blue) and stim conditions (shown in red) for participants with evoked responses to stimulation (t_{12} = 1.89, *p* = .083, *d* = .52, n=13). **(B)** Proportion of time spent in each sleep stage, relative to the total duration, participants as in A. Stimulation significantly reduced SWS and increased N2 proportion (t_{12} = 4.91, *p* < .001, *d* = 1.36 and W_{12} = 85, *p* = .003, *r* = .77, respectively). No differences were observed in N1 (W_{12} = 56, *p* = .497, *r* = .20) or REM (t_{12} = .50, *p* = .625, *d* = .14). **(C)** Sleep duration (in seconds) in sham (shown in blue) and stim conditions (shown in red) for participants without evoked responses to stimulation (t_6 = .48, *p* = .180, *d* = .48, n=7). **(D)** Proportion of time spent in each sleep stage, relative to the total duration, participants as in C. Stimulation significantly reduced SWS and increased N2 proportion (t_6 = 3.30, *p* = .016, *d* = 1.25 and t_6 = -3.45, *p* = .014, *d* = -1.31, respectively). Again, no differences were observed in N1 (t_6 = 1.16, *p* = .290, *d* = .44) or REM (W_6 = 9, *p* = .787, *r* = .16). **(E)** Declarative memory performance, shown as figural, verbal, and overall declarative memory score after the stimulation (red) and non-stimulation nap (blue), participants as in A. Stimulation significantly reduced the figural memory score (t_{12} = -3.51, *p* = .004, *d* = .97). No effects were observable for verbal memory (t_{12} = -.38, *p* = .711, *d* = .11) or the overall declarative memory score (t_{12} = -1.17, *p* = .264, *d* = .32). **(F)** Declarative memory performance, shown as figural, verbal, and overall declarative memory score after the stimulation (red) and non-stimulation nap (blue), participants as in C. Stimulation significantly reduced the figural memory score (t_6 = -2.46, *p* = .049, *d* = .93). No effects were observable for verbal memory (t_6 = -.05, *p* = .959, *d* = .02) or the overall declarative memory score (t_6 = -.66, *p* = .534, *d* = .25). Significance levels are denoted as follows: *** < .001, ** < .01, * < .05, + < .1. Violin plots show median values as black dot and quartiles as darker shaded area on the left side, as well as condition means of the individual participants as dots on the righthand side.

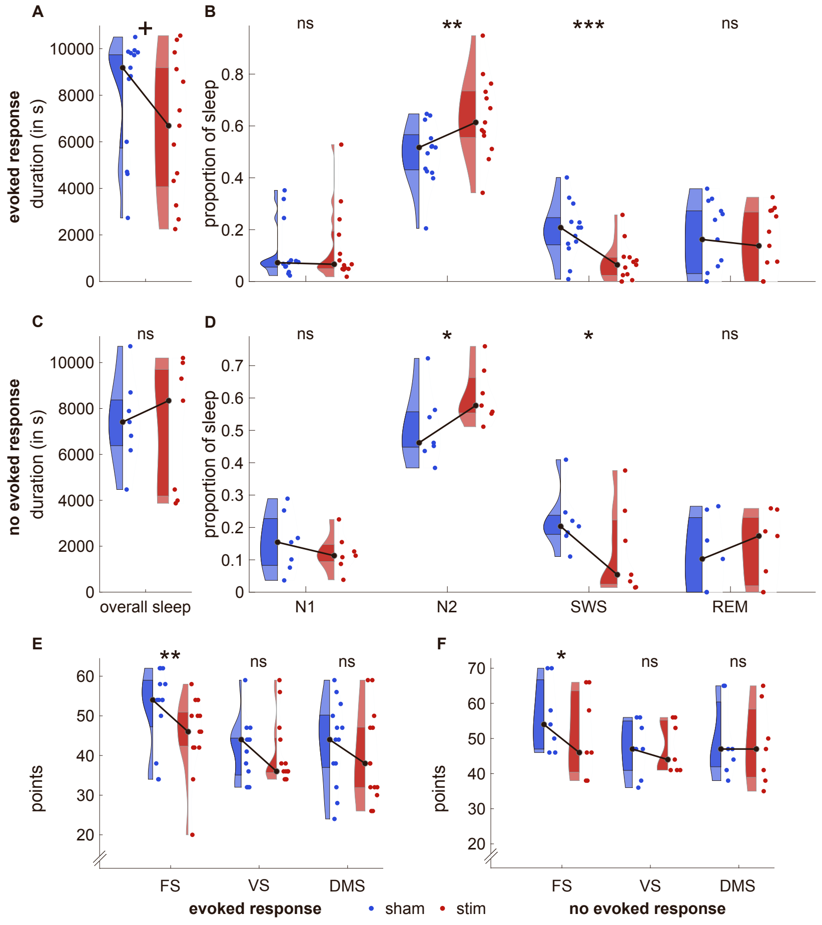

Supplementary Figure S3. Event-related potentials by reference type. ERP analyses were also computed using mastoid reference instead of the common average reference to establish that effects were not as a byproduct of referencing. A linked mastoid reference was created using channels TP9 and TP10, given that no mastoid channels were recorded. The lack of recorded mastoids as well as the comparable results with either reference type justified the choice of the common average reference used throughout. **(A)** Mastoid: Stimulation triggered a negative event-related potential (ERP) across all sleep stages. Plot displays average response post sound onset in significant cluster channels (Fp1, Fp2, F7, F3, Fz, F4, F8, FC5, FC1, FC2, FC6, T7, C3, Cz, C4, T8, CP5, CP1, CP2, CP6, P7, P3, Pz, P4, P8). Time of significant difference according to permutation-based statistics shown as red bar. **(B)** Common average reference: Stimulation triggered a negative event-related potential (ERP) across all sleep stages, however with lower potential overall. Plot displays average response post sound onset in significant cluster channels (Fp1, Fp2, F3, Fz, F4, FC5, FC1, FC2, FC6, C3, Cz, C4, CP5). Time of significant difference according to permutation-based statistics shown as red bar. Lines and shaded area denote mean ± SEM.

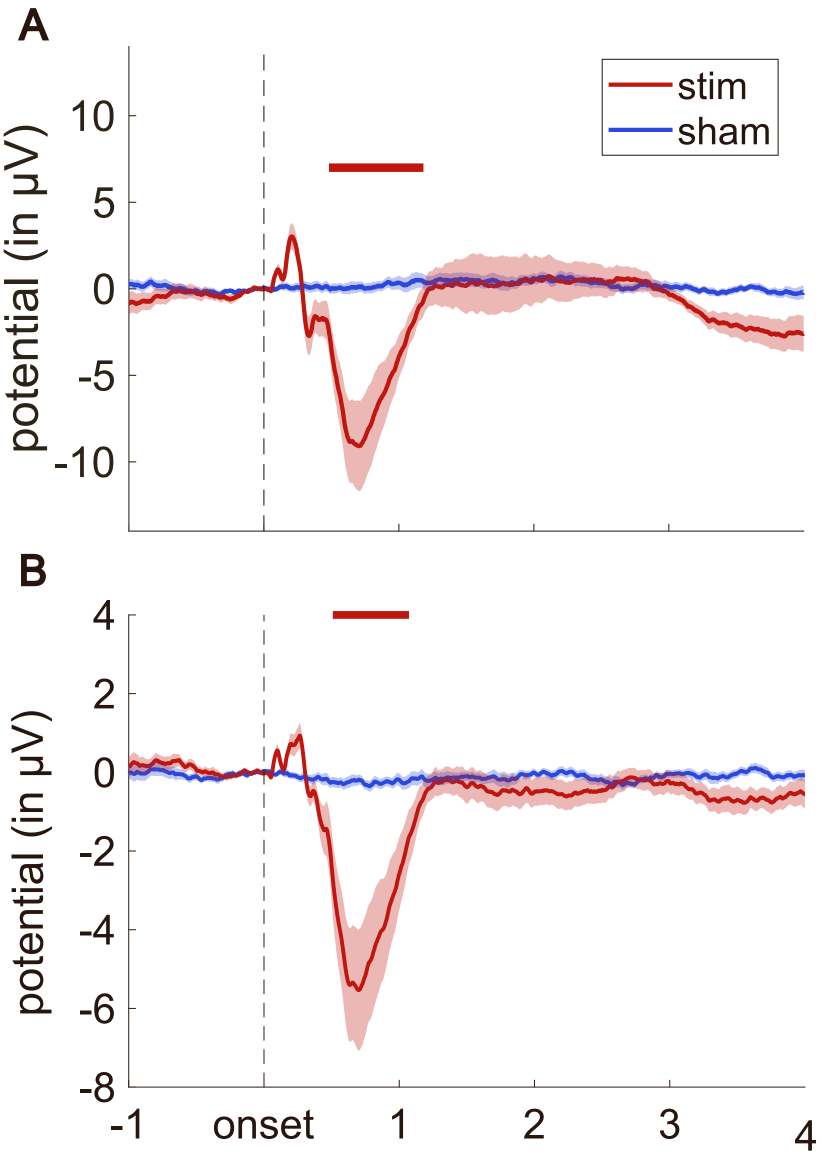

Supplementary Figure S4. Phase coupling of slow waves and sleep spindles in frontal and parietal channels. It should be noted that, due to the reduced number of identified slow waves in the stimulated condition, there were also fewer slow wave-spindle pairings identified across the ROIs in the stimulated (frontal: M =176.30, SE =42.56, parietal: M = 82.40, SE = 19.86) than in the sham condition (frontal: M = 198.60, SE = 45.18, parietal M = 86.60, SE = 25.50). However, this was not significant in both ROIs (Frontal: $t_{19} = -0.55$, $p = 0.56$; Parietal: $t_{19} = -0.16$, $p = 0.87$, n=20).

(A) Mean distribution of slow wave phase in which spindles originated in frontal channels (Fz, F3, F4) across participants. We used circular statistics, specifically the Hotelling paired sample test for equal angular means, to further compare stimulation (red) and sham (blue) conditions. The mean phase of slow wave-spindle pairs in the stimulated (*M* = 203.55°, *SE* = 7.44, *SD* = 33.27°) was not significantly different from the sham condition (*M* = 217.66°, *SE* =7.64°, *SD* = 34.18°, $F_{19} = 1.69$, p = 0.21). **(B)** Same as A but in parietal channels (Pz, P3, P4). There was no significant difference between the stimulated (*M* = 192.59°, *SE* =12.06°, *SD* = 53.94°) and sham condition (*M* = 189.65°, *SE* =12.11°, *SD* = 54.19°) in parietal regions ($F_{19} = 0.015$, $p = .98$).

(C) In frontal regions, auditory stimulation significantly increased the proportion of slow waves (in %) coupled with spindles (Stim: *M* = 10.41%, SEM = 1.81%, SD = 8.09%; Sham: *M* = 6.56%, SEM = 1.55%, SD = 6.92%; $t_{19} = 2.46$, $p = 0.024$, $d = 0.55$), indicating enhanced coupling efficiency. **(D)** Similarly, in parietal regions, stimulation significantly increased the proportion of coupled slow waves (Stim: *M* = 5.60%, SE = 1.22%, SD = 5.44%; Sham: *M* = 2.54%, SE = 0.60%, SD = 2.67%; $t_{19} = 3.32$, $p = 0.004$, $d = 0.74$). However, the absolute number of coupled slow waves did not differ significantly between conditions for frontal (**E:** Stim: *M* = 174.95, SEM = 42.09, SD = 188.29; Sham: *M* = 198.15, SEM = 44.98, SD = 201.16; $t_{19} = -0.58$, $p = 0.571$) or parietal channels (**F:** Stim: *M* = 82.20, SEM = 19.81, SD = 88.62; Sham: *M* = 86.35, SEM = 25.50, SD = 114.02; $t_{19} = -0.16$, $p = 0.875$). Horizontal black lines denote mean value, with the vertical lines indicating standard error of the mean. Dots denote mean condition values of individual participants.

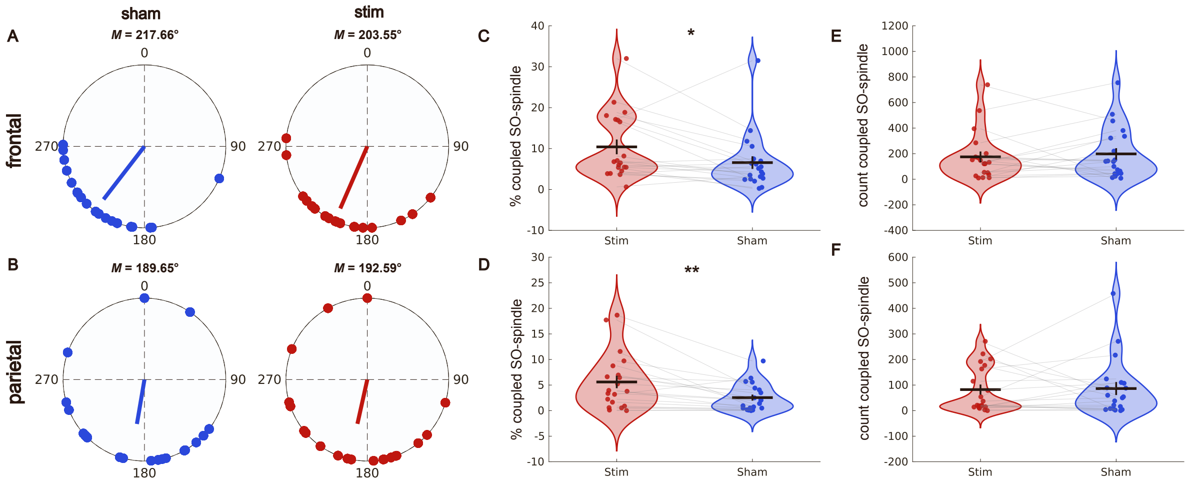

Supplementary Figure S5. No effects of auditory stimulation on motor sequence memory. In the procedural task (finger tapping), reaction time, the number of entered sequences, and the number of correctly entered sequences served as dependent measures and were each assessed by a 2 (time: pre vs post nap) x 2 (condition: stimulation vs sham) repeated measures ANOVA. Given that the task design did not include a wake control, it is unclear whether the differences in time were due to sleep, time that has passed, or due to a natural improvement in the task over time.

(A) Speed (in ms) of finger tapping is displayed for the encoding part (pre nap) and the retrieval part (post nap), as well as the difference score with larger values indicating improvement. Colored left-hand plots show these differences separated by stimulation condition (sham in blue, stimulation in red), and right-hand plots separated by session (first in dark gray, second in light gray). Overall faster reaction times were observed post rather than pre nap (pre: M = 247.40, SE = 13.17, post: M = 212.36, SE = 10.30; $F_{1,19}$ = 11.93, p = .003, η_p^2 = .39). There was no significant main effect of stimulation nor an interaction effect (stim: $F_{1,19}$ = .86, p = .364, η_p^2 = .04, interaction: $F_{1,19}$ = .14, p = .709, η_p^2 = .01). **(B)** Same as in A, but indicating how many sequences were typed out. More sequences were typed out post nap (pre: M = 20.58, SE = .70, post: M = 22.93, SE = .71; $F_{1,19}$ = 15.10, p < .001, η_p^2 = .44). There was no significant main effect of stimulation nor an interaction effect (stim: $F_{1,19}$ = 1.41, p = .250, η_p^2 = .07, interaction: $F_{1,19}$ < .01, p = .983, η_p^2 < .01). **(C)** Same as in A, but indicating how many accurate sequences were typed out. More accurate sequences were typed post rather than pre nap (pre: M = 18.46, SE = .69, post: M = 20.94, SE = .78; $F_{1,19}$ = 14.51, p = .001, η_p^2 = .43). There was no significant main effect of stimulation nor an interaction effect (stim: $F_{1,19}$ = .67, p = .423, η_p^2 = .03, interaction: $F_{1,19}$ < .01, p = .974, η_p^2 < .01). Some assumptions for running ANOVA were violated, so we separately also calculated the interaction effect by comparing the difference score in timing (pre-post) for each the stimulation and sham condition. Again, there was no significant effect of interaction this way for RT (**A:** t_{19} = .38, p = .709, d = .08), completed sequences (**B:** V_{19} = 88.5, p = .550), or accurate sequences (**C:** V_{19} = 86.5, p = .502). Equivalent analyses were performed contrasting first and second sessions, showing no effects of the session on procedural memory with regards to speed **(A)**, completed sequences **(B)**, and accurately completed sequences **(C)**. Significance levels are denoted as follows: *** < .001, ** < .01, * < .05, + < .1. Violin plots show median values as black dot and quartiles as darker shaded area on the left side, as well as condition means of the individual participants as dots on the righthand side.

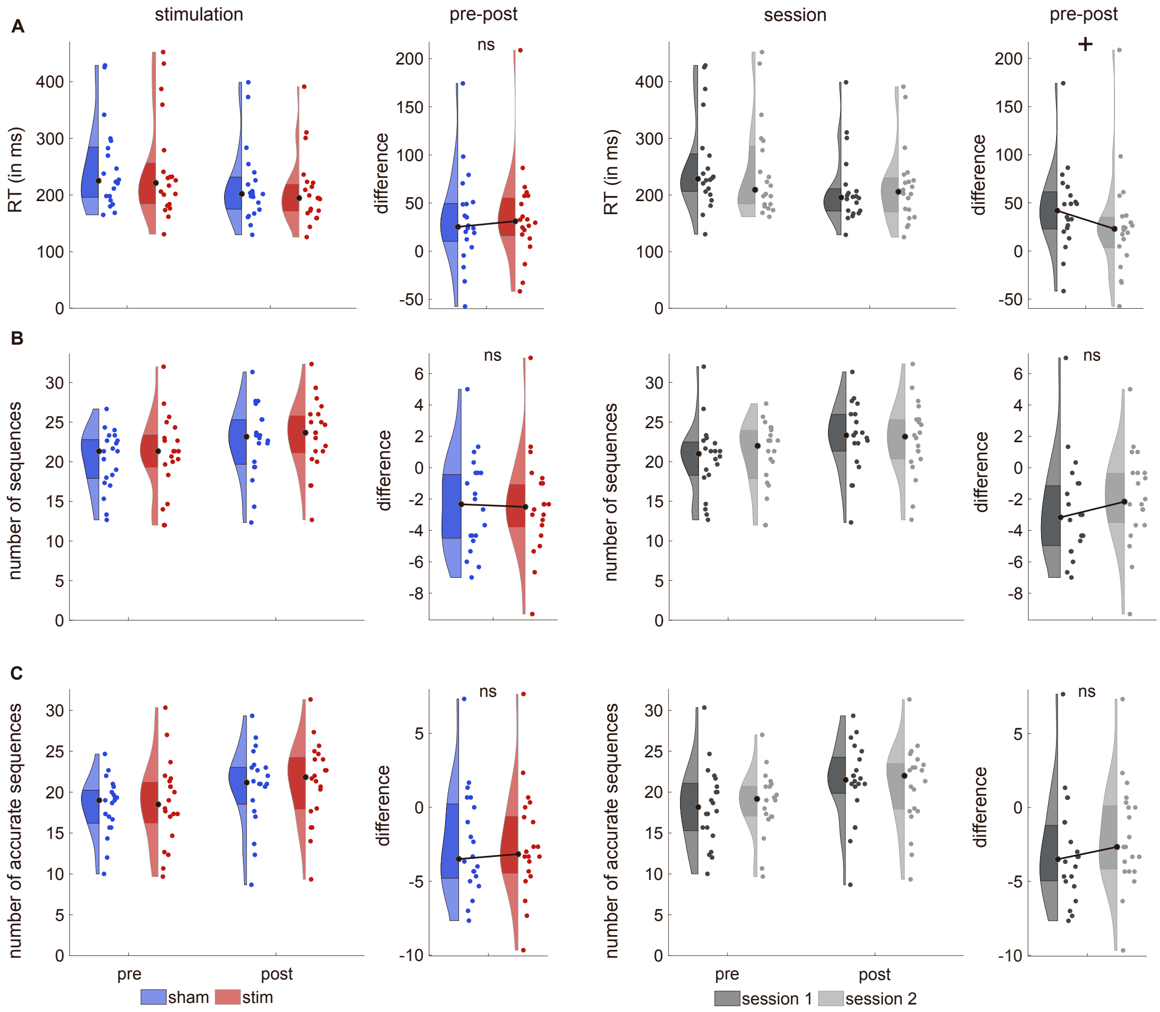

A
B
C
stimulation
pre-post
session
pre-post
ns
+
RT (in ms)
difference
number of sequences
number of accurate sequences
pre
post
sham
stim
session 1
session 2

Supplementary Figure S6. Effects of auditory stimulation on cortical spread of TSWs, swapped condition label randomization. Statistical significance for differences in cortical spread of TSWs between the stimulation and sham condition was additionally assessed by randomly swapping condition labels in participants. Observed values were compared with a random distribution created by randomly swapped condition labels (1000 times). **(A)** The condition difference is shown between the count of TSWs involving each sensor pair and illustrates the scalp-wide reduction in traveling waves in the stimulation condition. Data as in Figure 5A, significance from swapped-label randomization is indicated by green borders and mirrored along the diagonal axis. Purple color indicates larger differences, thereby relatively higher TSW count in the sham condition. **(B)** Difference in cortical spread of TSWs in each condition (as percent of TSWs involving each sensor pair) indicated broadly more scalp coverage for TSWs in sham (in blue), particularly in frontal sensors. Data as in Figure 5H, significance indicators by swapped-label randomization and mirrored along the diagonal axis. Blue tones denote more relative TSW engagement of the region in the sham condition, red more relative TSW engagement in the stim condition. Green signifies the observed value below 2.5% of the label-flipped distribution, black above 97.5%, in line with an alpha of .05. Sensors are arranged from right parietal over frontal to left parietal.

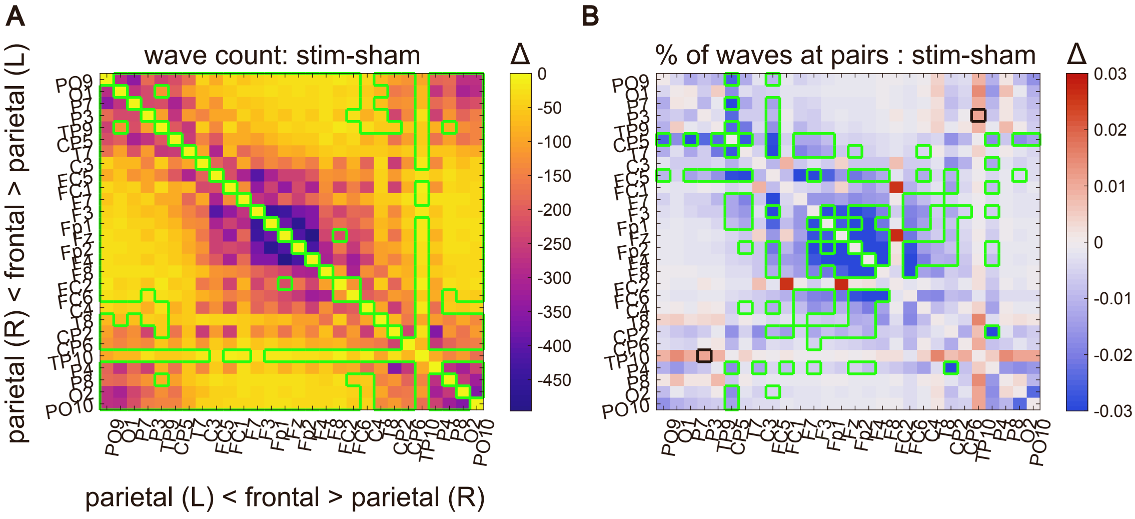

Supplementary Table S3. Linear mixed-effect models for the individual TSW features. Model estimates for fixed effects of relative time in SWS, the respective TSW feature, and stimulation condition (cond) are shown for the full model. Chi-squared statistics are shown for the improve in model fit (fit) when including the respective TSW feature in comparison to a reduced SWS-only model. All VIF values were below 2.3.

	**Peak duration**	**Size in electrodes**	**Maximal traveled distance**
**SWS**	β=.26, *SE*=.15, $t_{34.4}$=1.76, *p*=.087	β=.32, *SE*=.14, $t_{35.4}$=2.23, *p*=.033	β=.30, *SE*=.15, $t_{34.0}$=2.00, *p*=.054
**TSW**	β=.29, *SE*=.12, $t_{27.5}$=2.36, *p*=.026	β=.27, *SE*=.11, $t_{28.7}$=2.35, *p*=.026	β=.21, *SE*=.12, $t_{24.3}$=1.78, *p*=.088
**cond**	β=-.13, *SE*=.20, $t_{27.2}$=-.65, *p*=.523	β=-.13, *SE*=.20, $t_{27.7}$=-.63, *p*=.532	β=-.10, *SE*=.21, $t_{28.5}$=-.50, *p*=.624
**fit**	χ^2(1)=6.57, *p*=.010	χ^2(1)=6.50, *p*=.011	χ^2(1)=4.33, *p*=.037

Supplementary Table S4. Mediation analyses for the individual TSW features. Each model contained relative time in SWS as predictor, figural score as outcome, and the respective TSW feature as mediator. Confidence interval (CI) was over 95%. All VIF values were below 2.3.

	**Peak duration**	**Size in electrodes**	**Maximal traveled distance**
**a**	β = .50, *CI* = [0.139, 0.814], *p* = .003	β = .38, *CI* = [0.003, 0.697], *p* = .028	β = .48, *CI* = [0.132, 0.779], *p* = .002
**b**	β = .42, *CI* = [0.056, 0.756], *p* = .018	β = .39, *CI* = [0.020, 0.692], *p* = .024	β = .33, *CI* = [-0.116, 0.681], *p* = .106
**ab**	β = .21, *CI* = [0.007, 0.491], *p* = .089	β = .15, *CI* = [-0.016, 0.383], *p* = .153	β = .16, *CI* = [-0.049, 0.429], *p* = .191
**c**	β = .38, *CI* = [0.072, 0.620], *p* = .006	β = .44, *CI* = [0.173, 0.654], *p* < .001	β = .43, *CI* = [0.096, 0.702], *p* = .005
**total**	β = .59, *CI* = [0.284, 0.807], *p* < .001	β = .59, *CI* = [0.284, 0.807], *p* < .001	β = .59, *CI* = [0.284, 0.807], *p* < .001
